# Functional divergence of thyrotropin beta-subunit paralogs gives new insights into salmon smoltification metamorphosis

**DOI:** 10.1038/s41598-019-40019-5

**Published:** 2019-03-14

**Authors:** Mitchell S. Fleming, Gersende Maugars, Anne-Gaëlle Lafont, Jocelyn Rancon, Romain Fontaine, Rasoul Nourizadeh-Lillabadi, Finn-Arne Weltzien, Elena Santidrian Yebra-Pimentel, Ron Dirks, Stephen D. McCormick, Karine Rousseau, Patrick Martin, Sylvie Dufour

**Affiliations:** 1 Biology of Aquatic Organisms and Ecosystems (BOREA), Muséum National d’Histoire Naturelle, CNRS, IRD, Sorbonne Université, Université de Caen Normandie, Université des Antilles, 75231 Paris, Cedex 05 France; 2 Conservatoire National du Saumon Sauvage, 43300 Chanteuges, France; 3 Faculty of Veterinary Medicine, Norwegian University of Life Sciences, 0102 Oslo, Norway; 4 Future Genomics Technologies B.V, 2333 BE Leiden, Netherlands; 5 US Geological Survey, Leetown Science Center, Conte Anadromous Fish Research Laboratory, Turners Falls, MA USA

## Abstract

Smoltification is a metamorphic event in salmon life history, which initiates downstream migration and pre-adapts juvenile salmon for seawater entry. While a number of reports concern thyroid hormones and smoltification, few and inconclusive studies have addressed the potential role of thyrotropin (TSH). TSH is composed of a α-subunit common to gonadotropins, and a β-subunit conferring hormone specificity. We report the presence and functional divergence of duplicated TSH β-subunit paralogs (*tshβa* and *tshβb*) in Atlantic salmon. Phylogeny and synteny analyses allowed us to infer that they originated from teleost-specific whole genome duplication. Expression profiles of both paralogs in the pituitary were measured by qPCR throughout smoltification in Atlantic salmon from the endangered Loire-Allier population raised in a conservation hatchery. This revealed a striking peak of *tshβb* expression in April, concomitant with downstream migration initiation, while *tshβa* expression remained relatively constant. *In situ* hybridization showed two distinct pituitary cell populations, *tshβa c*ells in the anterior adenohypophysis, and *tshβb* cells near to the pituitary stalk, a location comparable to the pars tuberalis TSH cells involved in seasonal physiology and behaviour in birds and mammals. Functional divergence of *tshβ* paralogs in Atlantic salmon supports a specific role of *tshβb* in smoltification.

## Introduction

The Atlantic salmon (*Salmo salar*), like other salmonids, has a complex life cycle with the reproduction occurring in the upper part of the rivers, while the growth phase taking place in the ocean. A crucial life history transition, called smoltification (or parr-smolt transformation) initiates downstream migration and pre-adapts the juvenile salmon to seawater entry^1–3^. As recently reviewed^4,5^, smoltification may be viewed as a metamorphosis since it encompasses multiple morphological, physiological and behavioural changes allowing the transition from a life cycle stage and habitat to the next life stage in a different habitat (change of ecophase). Smoltification occurs during the juvenile stage, and therefore some authors refer to it as “second” or “secondary” metamorphosis in comparison to the larval metamorphosis that they refer to as “true” metamorphosis^6,7^. In amphibians, extensive research has demonstrated that larval metamorphosis is triggered mainly by thyroid hormones (TH, thyroxine T4 and triiodothyronine T3), the production of which is stimulated by a pituitary hormone, thyrotropin (or thyroid-stimulating hormone, TSH). TSH is comprised of two subunits, a common alpha subunit shared with the gonadotropins, luteinizing hormone and follicle-stimulating hormone, and a beta subunit (TSHβ) conferring hormone specificity^8^.

A histological study by Hoar first reported an activation of thyroid follicles in Atlantic salmon during smoltification^9^. An increase in T4 plasma levels was then shown during smoltification in various salmonids (coho salmon, *Oncorhynchus kisutch*^10,11^, masu salmon, *Oncorhynchus masou*^12^; Atlantic salmon^13^), even though changes in T4 plasma levels were relatively limited as compared to other hormones such as cortisol, insulin-like growth factor-1 and growth hormone^3,5^. Experimental hormonal treatments suggested that TH may be responsible for smoltification-related change in rheotaxis and swimming behaviour^14^ and olfactory imprinting^15^, but would be insufficient to induce preadaptation to osmoregulation in seawater^16^.

While many studies addressed TH, little is known on TSH and smoltification, and contradictory data have been obtained. In the Atlantic salmon, pituitary TSH cells were more numerous and had increased activity in presmolts and smolts than in parr^17^, but no ultra-structural changes of TSH cells were observed in coho salmon during smoltification^18^. No change or a slight decrease in pituitary *tshβ* mRNA levels were measured in smolts as compared to parr in Atlantic salmon^19,20^ and coho salmon^11^. No variations in pituitary and plasma TSH protein levels were reported in coho salmon throughout smoltification^11^.

While extant amphibians, birds and mammals possess only a single TSH (a single *tshβ* gene), recent studies revealed the presence of duplicated *tshβ* paralogs in some other vertebrates^21^. Duplicated *tshβ* genes originated from whole genome duplication events that occurred in early vertebrates (“1R/2R”, for “1^st^ and 2^nd^ rounds of whole genome duplication”) and in early teleosts (“3R”, for “3^rd^ round of whole genome duplication”)^21^. Thus, chondrichthyans, such as the elephant shark, *Callorhinchus milii*, and basal sarcopterygians, such as the coelacanth, *Latimeria chalumnae*, have two *tshβ* paralogs issued from 2R (*tshβ* and *tshβ2*), while tetrapods have lost the *tshβ2* paralog and conserved only a single *tshβ* gene^21^. *Tshβ2* has also been lost in the actinopterygian lineage, but various teleost species possess two *tshβ* paralogs as a result of 3R-duplication of *tshβ*^21,22^, named in the present study *tshβa* and *tshβb*, according to the most common nomenclature “a and b” for teleost 3R-paralogs. In the present study, we searched for *tshβ* paralogs in the Atlantic salmon, considering also the additional genome duplication that occurred in the salmonid lineage (“4R”)^23^.

We revealed the presence and expression of two *tshβ* paralogs in Atlantic salmon and brought new knowledge on the evolutionary history of *tshβ* genes in salmonids. We investigated the potential involvement of the *tshβ* paralogs in smoltification, using the Atlantic salmon from the Loire-Allier basin as a model. This population is the last extant salmon population able to migrate long rivers in Western Europe and is currently endangered. Samplings were performed at the “Conservatoire National du Saumon Sauvage” (CNSS), Chanteuges, France, whom breeds wild brood stock and produces juvenile salmon which are released at different developmental stages, as part of a conservation programme. Quality and timing of smoltification are key issues for this population, as smolts need to achieve a 900 km-downstream migration before reaching the Loire estuary in a narrow window of suitable physiological and environmental conditions. This requires new research advances on environmental and neuroendocrine regulatory mechanisms of smoltification and initiation of downstream migration. With the demonstration of a striking expression of one of the *tshβ* paralog, this study provides the first evidence of a peak expression of *tsh* during smoltification.

## Results

### Two thyrotropin beta subunit (*tshβ*) paralogs in the Atlantic salmon

Using the recent Atlantic salmon genome assembly (GCA_000233375.4)^24^, we retrieved two genes with two exons each, encoding complete TSH β-subunit sequences: one gene (named in the present study *tshβa*) located on the chromosome ssa22 and corresponding to the *tshβ* sequence previously isolated^19^, and the second gene (named in the present study *tshβb*) located on ssa15 and coding for another *tshβ*; this *tshβb* sequence encompassed two exons, as all vertebrate *tshβ*, and included a previously identified exon 1^21^. In order to assess that the two *tshβ* paralogs are transcribed, cDNA sequences were successfully cloned using pituitary RNA from Atlantic salmon sampled during the parr-smolt transformation. A partial *tshβa* mRNA sequence (401 bp) was cloned and its sequence shared 100% identity with already characterized *tshβ* (AF060566)^19^. A *tshβb* mRNA sequence (517 bp) including the full length CDS was cloned (MG948546, this study); it presented 100% identity with the corresponding predicted *tshβb* sequence in the genome. A third putative *tshβ* locus was identified on ssa12, corresponding to a second paralog of *tshβa*. This locus includes only exon 2 with a frameshift mutation resulting in an early stop codon. Therefore this locus was identified as a *tshβa*-pseudogene (Supplementary Fig. S1A). In order to confirm this pseudogene, we cloned and sequenced the genomic sequence using DNA extracted from testis of an Imsa River (Norway) Atlantic salmon. The cloning confirmed the loss of exon 1 and the presence of the deletion in exon 2 leading to an early stop codon (Supplementary Fig. S1B). In addition, using Illumina short reads from an on-going sequencing project of the genome from a Loire-Allier Atlantic salmon, we aligned short reads against the two Atlantic salmon *tshβa* loci. For the pseudogene, read alignments confirmed the sequence of exon 2 with the presence of the deletion leading to the early stop codon. These results support the conclusion that the second *tshβa* paralog in Atlantic salmon is a pseudogene and that this loss is a common feature of Atlantic salmon populations.

Comparison of TSHβ deduced amino-acid sequences (Supplementary Fig. S2) showed that Atlantic salmon paralogs TSHβa and TSHβb shared 31.5% identity and 47.5% similarity and that both have conserved the twelve cysteine residues that have been shown to be required for proper folding and functional activity of TSH in mammals^25^. Both paralogs also shared the typical N-glycosylation site conserved among vertebrate glycoprotein hormone beta subunits; in addition, Atlantic salmon TSHβb presented a second N-glycosylation site located between the two first cysteine residues, as with the other teleost TSHβb^21^.

### Phylogeny analysis of TSHβ

**Figure 1:**
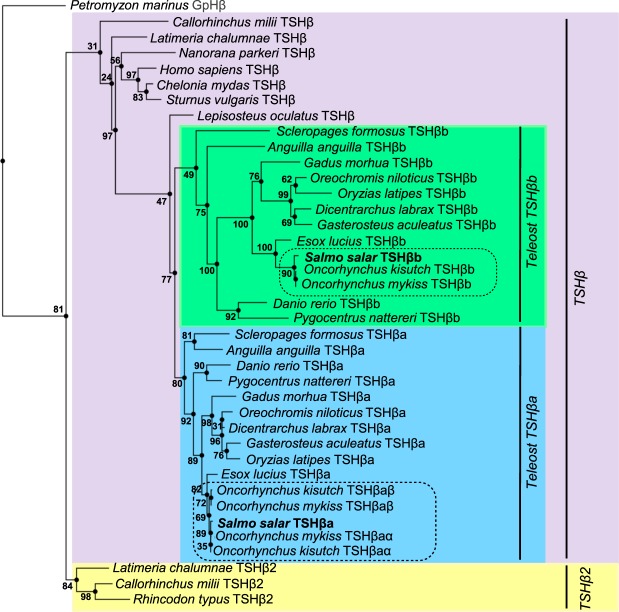
Consensus phylogenetic tree of TSHβ amino-acid sequences. Analysis was performed on 38 gnathostome TSHβ amino-acid sequences using the Maximum likelihood method, with 1000 bootstrap replicates. The tree was rooted using lamprey (*Petromyzon marinus*) GpHβ as outgroup. Bootstrap values are indicated at each node. The gnathostome TSHβ and TSHβ2 sister clades are highlighted in purple and yellow, and the teleost TSHβa and TSHβb sister clades, in blue and green, respectively. For sequence alignment, see Supplementary Fig. S2 and for sequence references, see Supplementary Table S1.

Molecular phylogeny analysis was performed on 38 gnathostome TSH β-subunit sequences and using lamprey, *Petromyzon marinus*, glycoprotein hormone β-subunit (GpHβ) as outgroup (Fig. 1; Supplementary Fig. S2). As previously shown^21^, most sequences grouped into a “classical TSHβ” clade, whereas a few chondrichthyan and basal sarcopterygian sequences formed a small TSHβ2 sister clade. Among the TSHβ clade, the analysis also supported the two sister clades for teleost sequences resulting from teleost 3R, named here TSHβa and TSHβb following the teleost 3R paralogs nomenclature (previously named TSHβ and TSHβ3^21^). The two Atlantic salmon TSHβ branched into the two teleost TSHβa and TSHβb clades, respectively (Fig. 1) allowing us to classify and name the Atlantic salmon TSHβ paralogs, TSHβa and TSHβb. The pike, *Esox lucius*, representative species of a sister group of Salmoniforms, the Esociforms which have not undergone the salmonid 4R, also possessed the two teleost *tshβ* 3R paralogs, encoding for TSHβa and TSHβb (Fig. 1). In contrast, we identified up to three *tshβ* genes in the genomes of rainbow trout, *Oncorhynchus mykiss* (GCA_002163495.1) and coho salmon (GCF_002021735.1): a single *tshβb* and two *tshβa* genes (*tshβaα* and *tshβaβ* according to the current nomenclature “*α* and *β*” for salmonid 4R paralogs^23^). They encode for three putative TSHβ (TSHβaα, TSHβaβ and TSHβb, Fig. 1, Supplementary Fig. S2).

### Synteny analysis of *tshβ* genomic region

**Figure 2:**
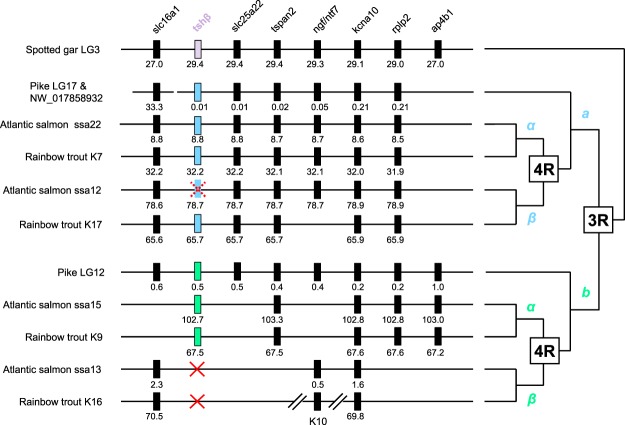
Synteny analysis of *tshβ* genomic region in actinopterygians. *Tshβ* genomic region of a non-teleost actinopterygian, a holostean, the spotted gar (*Lepisosteus oculatus*) was used as a reference. *Tshβ* genomic region was duplicated by teleost-specific whole genome duplication (3R) as seen in the pike (*Esox lucius*), resulting in two paralogons (a and b), and further duplicated by salmonid-specific whole genome duplication (4R) as seen in the Atlantic salmon (*Salmo salar*) and rainbow trout (*Oncorhynchus mykiss*), resulting in four paralogons (aα, aβ, bα, bβ). For each species, chromosome or scaffold number is indicated. Gene positions are given (in Mega base) below the genes. Full names and references of *tshβ* and neighbouring genes are given in Supplementary Table S2. Red cross indicates loss of *tshβ* paralog; dotted red cross indicates *tshβ* pseudogene.

To further assess the origin and nomenclature of salmonid *tshβ* paralogs, we performed a synteny analysis (Fig. 2) of the *tshβ* genomic region of the pike and of two salmonids, the Atlantic salmon and the rainbow trout. We used as a reference the *tshβ* genomic region of the spotted gar, *Lepisosteus oculatus*, holostean basal actinopterygian, which has not undergone the teleost 3R. Synteny analysis confirmed that the *tshβ* genomic region has been duplicated into two paralogons in the pike, in agreement with the teleost 3R, and further duplicated into four paralogons in the Atlantic salmon and rainbow trout, in agreement with the salmonid 4R. As an example, *kcna10*, one of *tshβ* neighbouring genes, was present as a single gene in the spotted gar, as two paralogs in the pike, and as four paralogs in the Atlantic salmon and rainbow trout, reflecting full conservation of 3R- and 4R-duplicated paralogs (Fig. 2). Other *tshβ* neighbouring genes, such as *rplp2*, *tspan*, or *slc16a1*, showed full conservation of 3R-duplicated paralogs, but incomplete conservation of 4R-duplicated paralogs leading to only three paralogs in salmonids (Fig. 2). Further paralog gene losses were observed for other neighbouring genes, and in particular for *ap4b1*, with a single gene present in the pike and salmonids, as in the spotted gar, indicating losses of 3R- and 4R-duplicated paralogs (Fig. 2). With regards to *tshβ*, synteny analysis confirmed that *tshβa* and *tshβb* paralogs arose from the teleost 3R, as shown in the pike (Fig. 2). Concerning *tshβa*, synteny analysis assessed that salmonid 4R further gave rise to *tshβaα* and *tshβaβ* paralogs, both conserved in the rainbow trout, while only *tshβaα* was conserved in the Atlantic salmon. Using Oxford Nanopore long reads from the on-going sequencing project of the genome from a Loire-Allier Atlantic salmon, we were able to further confirm the pseudogenization of *tshβaβ*. Long reads spanning the neighbouring genes of *tshβaβ* confirmed the lack of *tshβaβ* exon 1 and the presence only of *tshβaβ* exon 2 including the deletion leading to an early stop codon. For simplicity, in this study we named the *tshβaα* paralog conserved in the Atlantic salmon, *tshβa*. Concerning the 4R-duplicated *tshβb* paralogs, synteny supported that only one paralog was conserved in the rainbow trout and Atlantic salmon, named here *tshβb*.

### Differential tissue distribution of *tshβa* and *tshβb* transcripts in the Atlantic salmon

**Figure 3:**
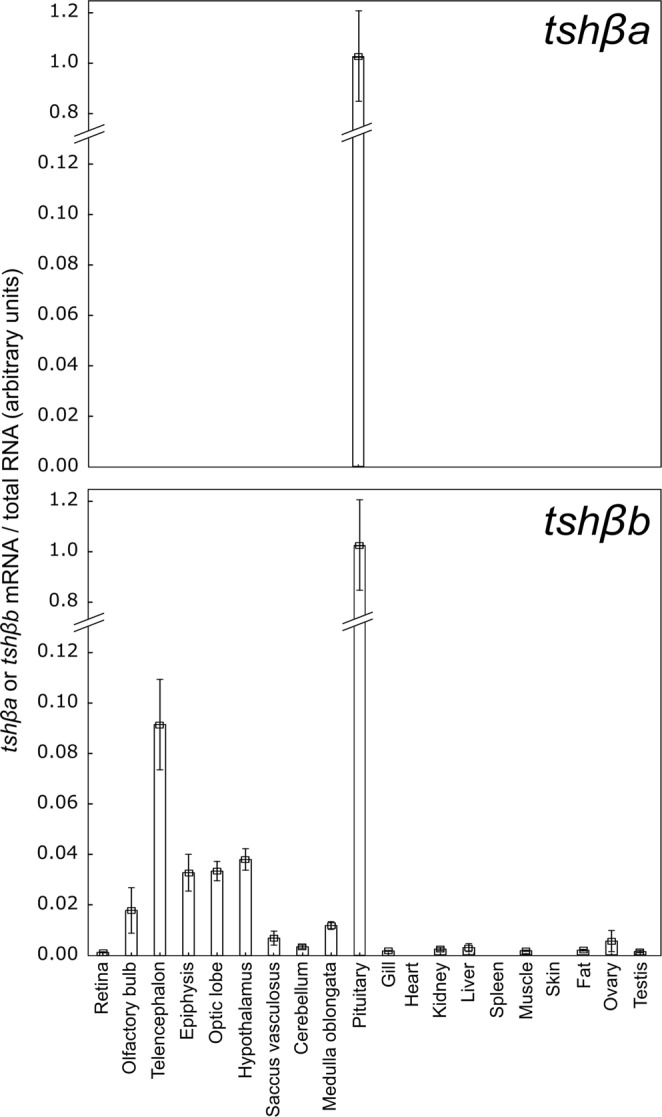
Tissue distribution of *tshβa* and *tshβb* transcripts in the Atlantic salmon. Messenger RNA levels of *tshβa* and *tshβb* paralogs were measured by qPCR in various tissues from smolts sampled in March 2015. Owing to the different nature of the tissues, transcripts levels were normalized to the amount of total RNA, and expressed as arbitrary units. Results are means ± s.e.m (n = 5 females for ovary; n = 5 males for testis; n = 10 mixed sex for the other tissues as there was no differences between sex).

We developed specific qPCRs for each Atlantic salmon *tshβa* and *tshβb* paralogs and compared the tissue distribution of their expression in smolts (Fig. 3). Both *tshβa* and *tshβb* paralogs were mainly expressed in the pituitary. While salmon *tshβa* transcript was exclusively found in the pituitary, *tshβb* was expressed also at low levels in various brain regions, and at lower but detectable levels in some peripheral tissues such as gills, kidney, liver, muscle, fat and gonads; *tshβb* transcripts were not detectable in heart, spleen and skin (Fig. 3).

### Evidence for the parr-smolt transformation during the experimental periods

Three independent experiments were performed in 2013, 2014 and 2016 where fish were sampled from December/January until June in order to cover the smoltification period that occurs in early spring for the Loire-Allier Atlantic salmon population^26^. Smoltification is classically described through typical morphological, behavioural and physiological changes. These series of changes were used to assess the occurrence of the smoltification.

#### Morphological changes

All juvenile salmon had visible parr marks at the first sampling time which progressively regressed throughout the parr-smolt transformation. Conversely, body silvering increased and darkening of the pectoral fins took place throughout the smoltification period. Photos were taken of each fish sampled in 2016 and representative photos are displayed in Supplementary Fig. S3. These changes are characteristic of the parr-smolt transformation (see review^3^).

#### Behavioural changes

Rheotactic behaviour, positive or negative, was observed every day during the daylight hours throughout the experimentations. All parr exhibited positive rheotaxis at the beginning of the experiments and maintained this behaviour until the end of March. The inversion of rheotaxis from positive to negative, typical of smoltification and which triggers the onset of downstream migration, was observed during early April in all experiments. All fish had inverted to negative rheotaxis by April 4 (2013), April 9 (2014) or April 8 (2016). They maintained negative rheotaxis until the end of the experimental period (end of June). This inversion timing in early April is in agreement with the previous reports for the Loire-Allier population^26^.

#### Physiological changes

**Figure 4:**
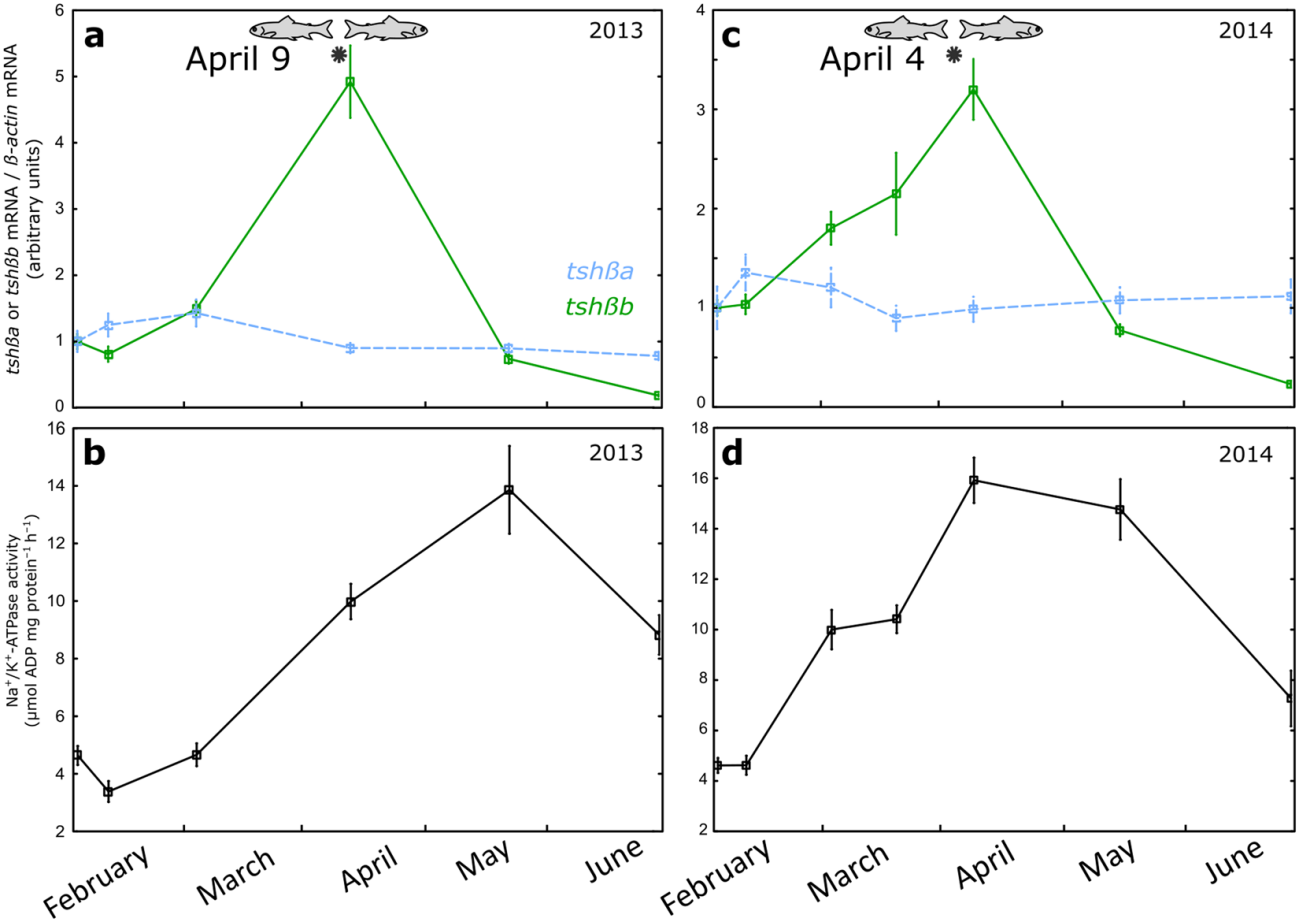
Profiles of pituitary *tshβa* and *tshβb* transcripts and of gill Na^+^, K^+^-ATPase (NKA) activity in the Atlantic salmon throughout the smoltification period (experiments 2013 and 2014). Two independent experiments were performed in 2013 and 2014. Under-yearling Atlantic salmon produced at CNSS were transferred in December, for each experimental year, to the experimental tanks under natural river water, temperature and photoperiod, and with circular water flow. Fish samplings were made from February to June. Pituitary messenger RNA levels of *tshβa* and *tshβb* paralogs were measured by qPCR, normalized to beta-actin as reference gene, and expressed as arbitrary units (**a**,**c**). Results are means ± s.e.m (2013 experiment, n = 20 individual pituitaries per sampling time; 2014 experiment, n = 10 pools of 2 pituitaries per sampling time). Gill NKA activity was measured according to^54^ and expressed as μmol ADP mg protein-1 h–1 (**b**,**d**). Results are means ±s.e.m (n = 10 fish per sampling time). Fish positive (fish facing the water current) or negative (fish facing downstream) rheotaxis was observed during daytime. *Indicates date of inversion of fish rheotaxis from positive to negative.

Gill Na^+^, K^+^-ATPase (NKA) activity was measured in fish sampled in 2013 and 2014 (Fig. 4). Gill NKA activity was low in parr (February), increased from March to reach a peak in smolts in April/May and decreased in post-smolts in June. The increase of gill NKA activity is a typical physiological characteristic of smoltification which prepares smolts to transition from fresh water to sea water (for review^3^).

### Peak expression of pituitary *tshβb* paralog during smoltification

In 2013 and 2014 fish were sampled from February to June. Pituitary *tshβa* and *tshβb* mRNA levels were measured by qPCR. While the expression profile of *tshβa* remained relatively constant throughout the sampling period, a dramatic peak in the expression of the other paralog, *tshβb*, was measured in March-April, during the smoltification period (Fig. 4). Notably, the *tshβb* expression peak was concomitant with the timing of rheotaxis inversion in both experiments (Fig. 4).

**Figure 5:**
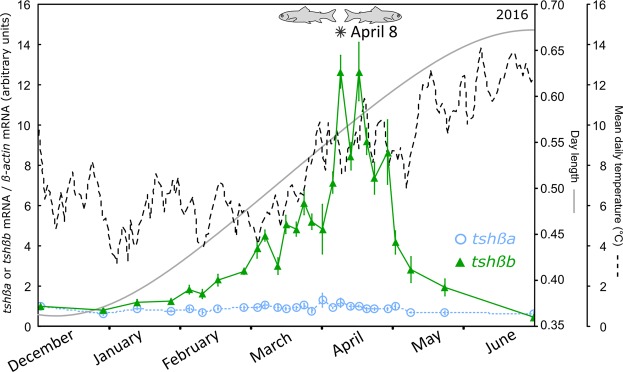
Profile of pituitary *tshβa* and *tshβb* transcripts in the Atlantic salmon throughout the smoltification period (experiment 2016). Under-yearling Atlantic salmon produced at CNSS were transferred in December to the experimental tanks under natural river water, temperature and photoperiod, and with circular water flow. Frequent fish samplings were made from December 2015 to June 2016. Messenger RNA levels of *tshβa* and *tshβb* paralogs were measured by qPCR, normalized to beta-actin as reference gene, and expressed as arbitrary units. Results are means ± s.e.m (n = 8 individual pituitaries per sampling group). Photoperiod and mean daily water temperature are indicated. Fish swimming behaviour and rheotaxis were observed during daytime. Fish positive (fish facing the water current) or negative (fish facing downstream) rheotaxis was observed during daytime. *Indicates date of inversion of fish rheotaxis from positive to negative.

In order to confirm the differential regulation of the pituitary expression of *tshβa* and *b* paralogs, we performed a third experiment from December 2015 to June 2016, with high frequency sampling during the smoltification period (Fig. 5). The results were in full agreement with 2013 and 2014 experiments. Pituitary expression of *tshβa* remained stable throughout the experiment, while a large expression peak of *tshβb* was recorded during the smoltification period. Pituitary *tshβb* transcript levels started to rise during February, reached a dramatic peak in early April and then dropped at the end of April until June, to reach lower levels than in December (Fig. 5). It is noteworthy that qPCR results (Figs 4 and 5) were expressed as arbitrary units for each paralog; however comparison of quantification cycle values (Cq) between paralogs suggested lower pituitary levels of *tshβb* than *tshβa* at the beginning (December/February) and end (June) of the experiments (8 Cq mean difference), and even in April at the time of *tshβb* peak (4 Cq mean difference). We observed again that the change in rheotaxis from positive to negative, a characteristic of smoltification, was concomitant with the expression peak of *tshβb* paralog (Figs 4 and 5).

### Distinct populations of *tshβa*- and *tshβb*-expressing cells in the Atlantic salmon pituitary

**Figure 6:**
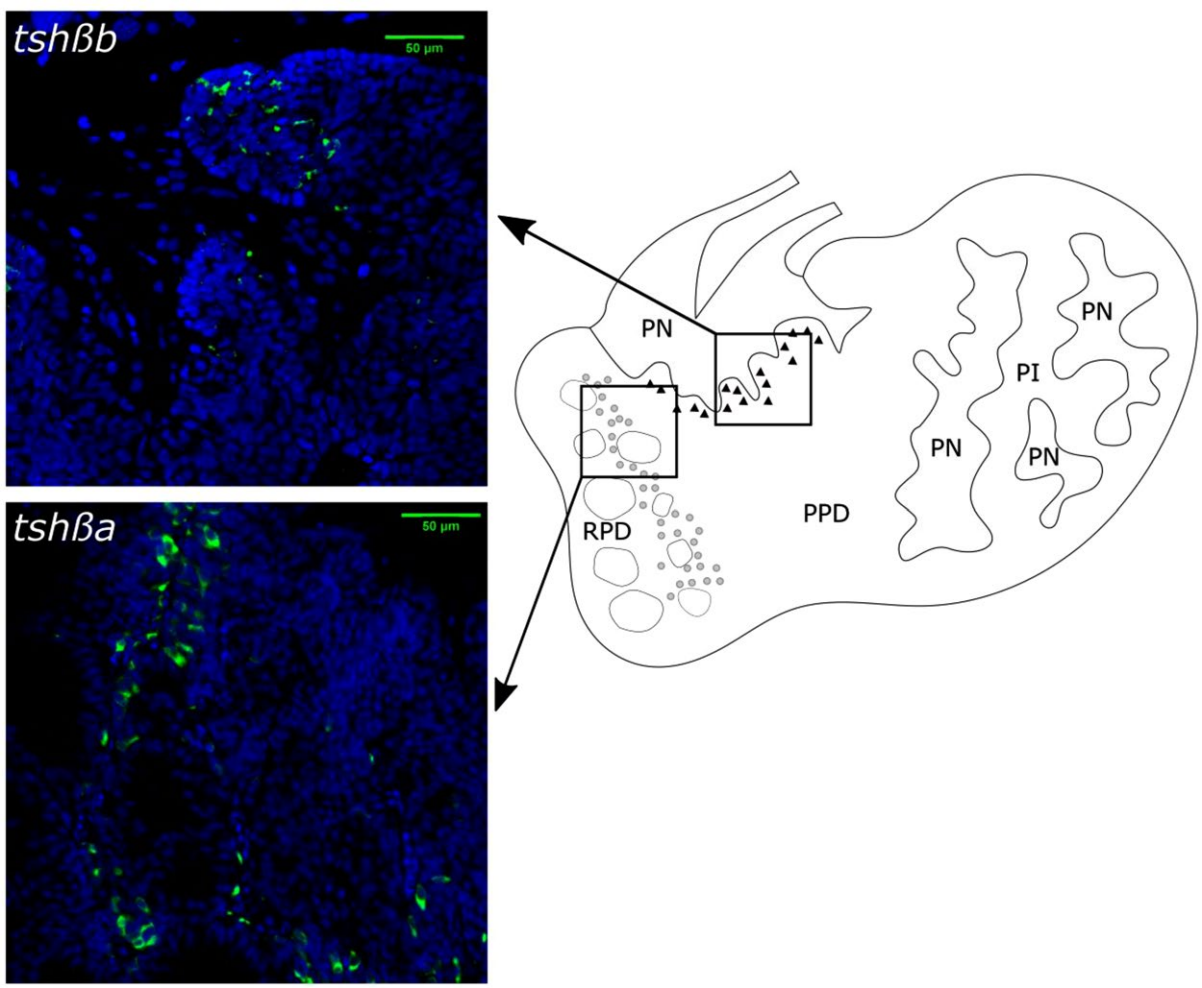
Localization by FISH of *tshβa* and *tshβb* transcripts in the Atlantic salmon pituitary. Fluorescent *in situ* hybridization (FISH) of *tshβa* and *tshβb* was performed on 70 µm parasagittal sections of pituitaries of smolts sampled in April, 2017. FISH photos: *tshβa* and *tshβb* cells are labelled in green (FITC); cell nuclei are labelled in blue (DAPI); upper: *tshβb* labelling: Confocal Z-projection from 3 µm Z-stack; lower: *tshβa* labelling: Confocal Z-plan image. Diagram: representation of the localization of *tshβa*- and *tshβb*-expressing cell populations; *tshβa*-expressing cells are located in the rostral pars distalis (RPD) close to the prolactin follicles (grey circles), at the border with the proximal pars distalis (PPD); *tshβb*-expressing cells are less numerous and located in the dorsal PPD close to the pars nervosa (PN) of the pituitary stalk (black triangles). No FITC labelling was observed in the pars intermedia (PI). Controls were performed using FITC sense probes and showed no labelling (see Supplementary Fig. S4).

In order to get more insight into the pituitary expression of the two paralogs, we compared the localization of *tshβa* and *tshβb* transcripts by fluorescent *in situ* hybridization (FISH), using pituitaries from parr sampled in October and November, smolts sampled in April and post-smolts in June. *Tshβa***-**expressing cells could be detected by FISH in parr and in smolts at all sampling times, while *tshβb***-**expressing cells could be observed only in April. The lack of detection of *tshβb***-**expressing cells by FISH in parr and in post-smolts is in agreement with the very low expression of this paralog outside of the smoltification period, as shown by qPCR. *Tshβa* cells were located in the anterior adenohypophysis, in the rostral pars distalis (RPD) close to the follicles formed by the prolactin cells, at the border with the proximal pars distalis (PPD) (Fig. 6). In contrast, *tshβb* cells were observed in the dorsal region of the PPD, bordering the dorsal region of the pars nervosa (PN) near the pituitary stalk (Fig. 6). No labelling was observed when using control sense probes (Supplementary Fig. S4). FISH results demonstrated that salmon *tshβa* and *tshβb* paralogs are expressed by two distinct cell populations within the pituitary.

## Discussion

The present study revealed that two paralogous genes, named *tshβa* and *tshβb* based on phylogeny and synteny analyses, are expressed in the Atlantic salmon. The deduced protein sequences TSHβa corresponded to the subunit previously characterized in Atlantic salmon^19^, while TSHβb corresponded to a novel not yet investigated subunit. A third homologous gene sequence was identified as a pseudogene. Atlantic salmon TSHβa and b sequences shared the 12 cysteine residues and the N-glycosylation site conserved among vertebrate TSHβ. Yet, they were largely different with only 47.5% amino-acid similarity reflecting their divergence since the teleost 3R genome duplication event. Differently to TSHβa, Atlantic salmon TSHβb possessed a second glycosylation site, as previously observed for other teleost TSHβb^21^, which may confer differential biological properties. Recent work in mice has shown that tissue specific glycosylation of TSH, produced in pars distalis *versus* in pars tuberalis, induces differential bioactivity in the blood^27,28^.

**Figure 7:**
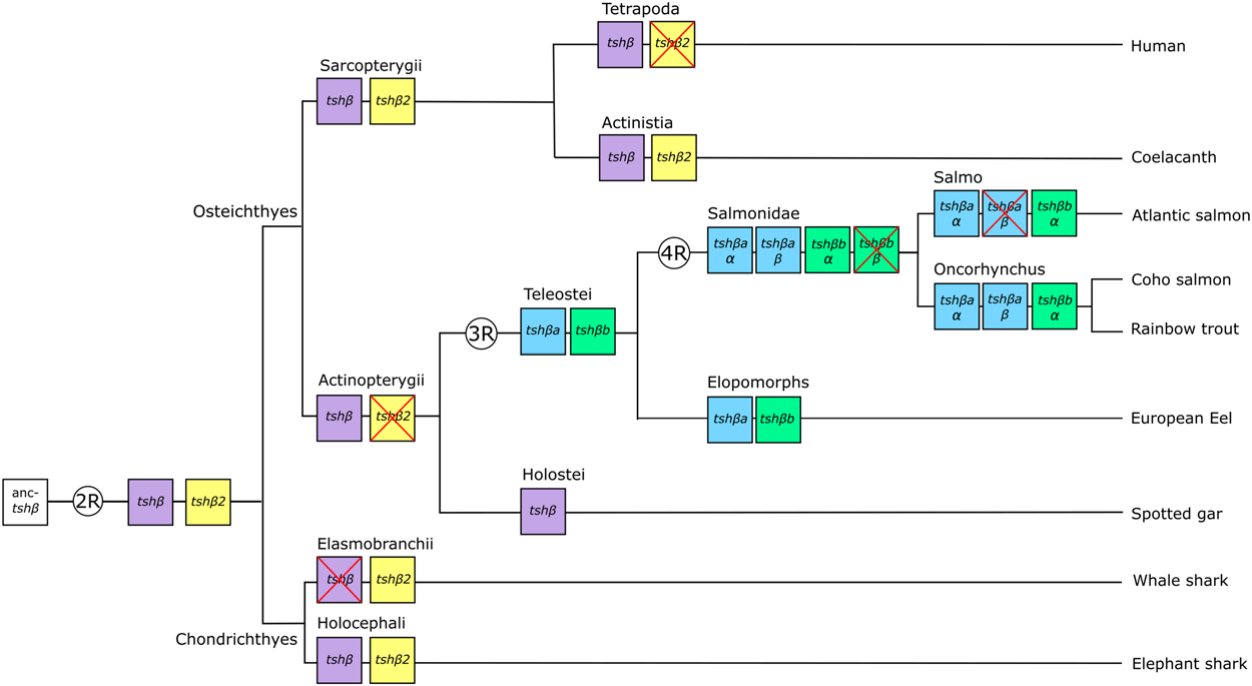
Proposed evolutionary scenario of vertebrate *tshβ* with a special focus on salmonids. The evolutionary scenario is based on phylogeny and synteny analyses^21^ (and the present study). Examples of extant representative species are listed on the right. *Tshβ* and *tshβ2* arose from the 2^nd^ round of whole genome duplication in early vertebrates (2R). *Tshβ2* paralog was lost early in the actinopterygian lineage. *Tshβa* and *tshβb* arose from the teleost-specific 3^rd^ round of whole genome duplication (3R) and were further duplicated by the salmonid-specific 4^th^ round of whole genome duplication (4R). One of the 4R paralogs , *tshβbβ*, was lost early in the salmonid lineage, before the divergence between the *Oncorhynchus* and *Salmo* lineages. Another 4R paralog, *tshβaβ*, still present in the rainbow trout (*Oncorhynchus mykiss*) and coho salmon (* Oncorhynchus kisutch*), is undergoing a loss in Atlantic salmon (*Salmo salar*) where it is detected as a pseudogene.

Phylogeny and synteny analyses brought new advances on the evolutionary scenario of *tshβ* genes, as illustrated in Fig. 7. As previously indicated^21^, the duplicated paralogs *tshβ* and *tshβ2* originated from vertebrate 2R, but only *tshβ* was conserved in the actinopterygian lineage as observed in an extant holostean, the spotted gar. Teleost 3R duplicated *tshβ* into *tshβa* and *tshβb*, both conserved in various extant teleosts. Salmonid 4R further duplicated *tshβa* into *tshβaα* and *tshβaβ* paralogs, both conserved in *Oncorhynchus* species; however, *tshβaβ* is undergoing a loss in Atlantic salmon, where it was detected as a pseudogene. We confirmed the *tshβaβ* pseudogene sequence using representatives from two wild Atlantic salmon populations (Loire-Allier, France and Imsa, Norway). This indicates that *tshβaβ* loss is a common feature among Atlantic salmon and does not represent a divergence related to domestication of the strain^24^ used for the published genome. Salmonid 4R would have also duplicated *tshβb* into two paralogs, but a single *tshβb* gene has been conserved in the *Oncorhynchus* and *Salmo* species investigated in this study; this suggests that one of the *tshβ* 4R paralogs would have been lost in the salmonid lineage, shortly after the 4R, before the divergence between *Oncorhynchus* and *Salmo* species (Fig. 7).

Development of specific qPCR for Atlantic salmon *tshβa* and *tshβb* allowed us to show that both genes are mainly expressed in the pituitary, and suggested that *tshβa* transcript levels are higher than *tshβb* transcript levels. The expression of both *tshβa* and *tshβb* paralogs in the pituitary has been previously reported in two other teleost species, the stickleback, *Gasterosteus aculeatus* (named *tshβ1* and *tshβ2* by the authors^22^) and the European eel, *Anguilla anguilla* (named *tshβ* and *tshβ3* by the authors^21^).

As measured by qPCR in the Atlantic salmon, *tshβa* was exclusively expressed in the pituitary, while *tshβb* was also expressed at lower levels in different brain regions, and in various peripheral tissues. A similar result was previously obtained in the European eel, with a pituitary-only expression of *tshβa* and a more ubiquitous tissue distribution of *tshβb (tshβ* and *tshβ*3 respectively^21^). In amphibians, reptiles and birds, which possess a single *tshβ* gene, most studies have reported the expression of *tshβ* mRNA only in the pituitary (chicken^29^; bullfrog^30^; quail^31^; turtle^32^; duck^33,34^) while investigating a wide range of other tissues (brain, gonads, liver, thyroid, muscle, lung, heart, intestine, kidney and spleen). However in mammals, which also possess a single *tshβ* mainly expressed in the pituitary, *tshβ* transcript or TSH protein were reported at low levels in the brain^35^ and in some peripheral tissues, such as myometrium and skin^36^.

We further investigated by FISH the respective localization of *tshβa* and *tshβb* in the Atlantic salmon pituitary and revealed that the paralogs were expressed by distinct pituitary cell populations. Numerous *tshβa*-expressing cells were located at the border between the RPD and antero-ventral PPD, while fewer *tshβb*-expressing cells were observed in the dorsal PPD close to the pituitary stalk. Early histological works in salmonids using radiothyroidectomy already reported the localization of TSH cells mainly in the RPD at the junction with PPD (Atlantic salmon^37^; chinook salmon, *Oncorhynchus tshawytscha*^38^). Immunocytochemical studies, using an antibody against human TSHβ, revealed TSH cells in the ventral PPD adjacent to the RPD (chum salmon, *Oncorhynchus keta* and rainbow trout^39,40^). A similar localization was observed using an antibody raised against purified coho salmon TSH (rainbow trout^41^; chinook salmon^42^). In light of our present study, these previous investigations likely observed the localization of the abundant TSHβa.

Two distinct populations of TSH cells have also been described in the pituitary of birds and mammals, but both expressing the same single gene present in tetrapods (*tshβ*). The “classical” TSH cell population is located in the pars distalis (PD), while a less numerous TSH cell population is located in the pars tuberalis (PT) which surrounds the pituitary stalk (quail^43^; Soy sheep^44^; mice^45^; European hamster^46^). We may relate the localization of salmon *tshβa-* and *tshβb-*expressing cells to that of amniote PD- and PT-*tshβ* cells, respectively, and infer that the specific expression of *tshβa* and *tshβb* in these distinct pituitary cell populations would represent a typical case of subfunctionalization of duplicated paralogs.

The potential involvement of *tshβa* and *tshβb* paralogs in smoltification was investigated by measuring their pituitary expression profiles in juvenile Atlantic salmon from the Loire-Allier basin. Three independent sampling experiments were performed in 2013, 2014 and 2016 at CNSS. Classical smoltification-related changes such as body colouration, rheotaxis inversion and increased gill NKA activity were observed which indicate that these fish have undergone complete smolt development. Remarkably, a striking peak in the expression of *tshβb*, with no change in *tshβa*, was recorded in April, at the period of smoltification, in each yearly experiment. This is the first demonstration of a surge in pituitary *tshβ* in relation to smoltification metamorphosis in salmonids. In contrast, previous studies reported slightly lower^11,19^ or no change^20^ in pituitary *tshβ* transcript levels during smoltification; these investigations were in fact targeting the *tshβa* paralog, the expression of which remains relatively stable as shown in the present study. The demonstration of a differential regulation of the pituitary expression of salmon *tshβa* and *tshβb* paralogs, with a specific peak of *tshβb*, revealed a marked functional divergence of the two paralogs, conferring a specific role in smoltification to *tshβb* paralog.

**Figure 8:**
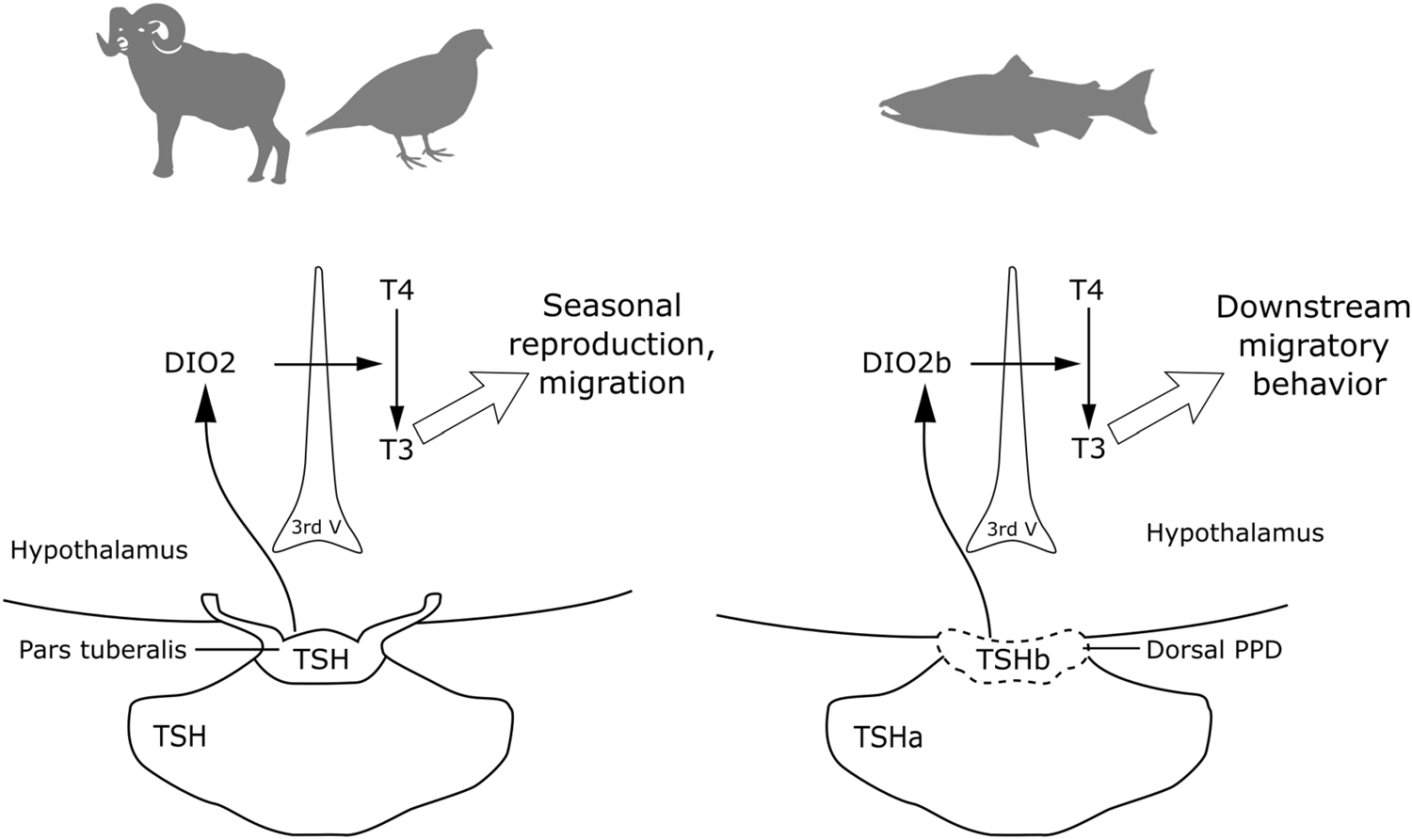
Proposed subfunctionalization of TSHa and TSHb, with potential role of TSHb in Atlantic salmon smoltification. In birds and mammals, the same gene (*tshβ*) is expressed by the “classical” TSH cells of the adenohypophysis and by the fewer TSH cells from the pars tuberalis (PT). PT-TSH is involved in the photoperiodic regulation of seasonal life traits^43,44,47,48^ by increasing brain deiodinase 2 (DIO2) and stimulating the conversion of thyroxin (T4) into triiodothyronine (T3). Salmon *tshβa* and *tshβb* paralogs are expressed by distinct cell populations as shown by FISH. We propose that *tshβb* cells located in the dorsal region of the pars proximal distalis (PPD) may be related to the PT-TSH cells in birds and mammals, and infer a subfunctionalization of salmon *tshβa* and *tshβb* paralogs. We revealed a peak expression of *tshβ* at smoltification, concomitant to the inversion of rheotaxis initiating downstream migration. An increase in brain *dio2b* paralog was reported at smoltification in Atlantic salmon^49^ and we hypothesize that the peak expression of *tshβb* may be responsible for this activation. Salmon TSHb may play a key-role in the environmental and internal regulation of smoltification and downstream migration, via similar signalling pathway as bird and mammal PT-TSH.

In birds and mammals, TSH produced by PT cells plays a key role in the seasonal regulation of major steps of life cycles, including reproduction, migration and hibernation^44,47,48^. PT-TSH stimulates the expression of type 2 deiodinase (DIO2), which catalyses the conversion of T4 into the more biologically active T3, thus leading to the activation of TH-regulated brain functions (Fig. 8). In a recent study^49^, a salmonid specific 4R-issued DIO2 paralog (*dio2b*) has been identified in the Atlantic salmon, the expression of which increases in circumventricular brain area of cell proliferation, during experimental photoperiod-induced smoltification. The authors proposed a specific role of DIO2b in promotion of TH-dependent brain development during smoltification^49^.

In the line of PT-TSH role and action mechanism in amniotes, we propose that TSHb produced by dorsal PPD cells in Atlantic salmon may stimulate brain DIO2b expression and promote TH-activated brain functions related to smoltification (Fig. 8). As a support to this hypothesis, in our three yearly experiments, the expression peak of *tshβb* paralog occurred simultaneously with smoltification-related changes in rheotaxis, which triggers the onset of downstream migration.

In the Atlantic salmon, *tshβb* was expressed not only in the pituitary but also at lower levels in various brain regions, so that additional regulatory pathways may also occur locally in some brain regions. Such a local signalling between light sensors, TSH and DIO2, has been proposed in the *saccus vasculosus* for the photoperiodic regulation of reproduction in masu salmon^50,51^. In our study, *tshβb* transcripts were detectable in the *saccus vasculosus* of the Atlantic salmon smolt but were at least 100 times less expressed than in the pituitary; *tshβa* transcripts were not detectable outside the pituitary. The expression of *tshβb* paralog, not only in the pituitary, but in various brain regions and peripheral tissues observed in the present study warrants future investigations on the possible variations of *tshβb* transcripts in these regions during smoltification.

In mammals, PT-TSH has been suggested to act not only on the basal hypothalamus but also to exert some paracrine effects in the pituitary^52^, so that we may also hypothesize pituitary actions of *tshβb* paralog during salmon smoltification. Using a basal teleost, the European eel, as a model, we previously identified 3R-duplicated TSH receptor (TSHR) paralogs and revealed the specific expression of *tshrb* paralog in various brain regions as well as in the pituitary, supporting brain and pituitary actions of TSH in teleosts^21^. Further studies should aim at investigating TSH receptor paralog number and tissue distribution in the Atlantic salmon.

In the present study, we propose a specific role of Atlantic salmon *tshβb* paralog at smoltification, related to the change in rheotaxis and triggering of downstream migration. The remarkable expression peak of pituitary *tshβb* demonstrated in our study may be of high relevance for deciphering the internal and environmental regulation of salmonid smoltification and initiation of downstream migration; especially concerning the endangered long-river Loire-Allier salmon population, understanding these mechanisms may provide new basis for its conservation. A specific role in migration of *tshβb* paralog (named *tshβ2* by the authors^22^) was also suggested in the stickleback^22^, based on higher pituitary transcript levels of *tshβb* in populations migrating to the sea, as compared to stream-resident populations, with no difference in *tshβa* paralog (named *tshβ1* by the authors^22^) between the two ecotypes. The authors suggested that genetic differences in cis-regulatory regions of *tshβb* gene may convey this adaptive divergence in migratory behaviour during stickleback radiation^22,53^. Similarly, there is large diversity of migration strategies among salmonid species and populations, from long-river anadromous to landlocked. Our present findings open new research avenues for comparing *tshβb* expression during smoltification, as well as *tshβb* regulatory genomic sequences, between salmonid species and ecotypes.

In conclusion, two *tshβ* paralogs are expressed in the Atlantic salmon by distinct pituitary cell populations, and exhibit a striking functional divergence, with a large expression peak of *tshβb*, but not of *tshβa*, during smoltification. This is the first demonstration of a peak expression of *tshβ* in salmonid smoltification. This involvement of the thyrotropic axis complies with the endocrine regulation of vertebrate metamorphosis typically observed in amphibian and flatfish. A specific role of *tshβb* paralog is suggested in the onset of smoltification-related downstream migratory behaviour, possibly mediated by the stimulation of brain DIO2 and T3 production, as shown for PT-TSH involved in the seasonal regulation of life cycle traits in birds and mammals. The remarkable functional divergence of *tshβa* and *tshβb* in salmon may have represented selective forces for the conservation of these duplicated paralogs.

## Methods

### Fish

The study was carried on juvenile Atlantic salmon (*Salmo salar*) of the Loire-Allier population raised indoor under natural water, temperature, and photoperiod conditions, at the Conservatoire National du Saumon Sauvage (CNSS), Chanteuges, France (Agreement N° B43 056 005; according to the ARRETE N° DDCSPP/CS/2016/40), which breeds wild returning adult Atlantic salmon genitors caught at the Vichy dam, 620 km from the Loire estuary. The research project was performed in accordance with guidelines and regulations according to the protocol approved by Cuvier Ethic Committee France.

For each experimental year, 390 under-yearling fish were transferred in December into two circular tanks (3 m diameter; depth range 0.5 m) supplied with UV filtered natural running water from the Desges River (tributary of the Allier). Experiments were conducted from December to end of June, which spans the smoltification period that occurs in early spring for the Loire-Allier Atlantic salmon population^26^. Three independent experiments were performed (2013, 2014 and 2016). Water temperature was measured using probes (Johnson control, Colombes, France; TS 9101: accuracy ±0.2 °C). An anti-clockwise flow was achieved by a tangentially oriented water inlet at the periphery of the tank and a central drain as previously described^26^. Photoperiod regime mimicked the natural photoperiod by using an outside light sensor that controlled the light above each tank. Each tank had a LedBulb (D 14–75 W E27 827 A67; Philips, Amsterdam, Netherlands) 3 m above the water surface. Fish were fed automatically with a custom fish diet (Turbot label Rouge, Le Gouessant, Lamballe, France) in excess five times a day at equal intervals during daylight hours. Fish swimming behaviour was visually observed during daytime: positive rheotaxis for fish facing the water current *versus* negative rheotaxis for fish swimming with the current^26^.

### Tissue collection

Fish were anesthetized with an overdose of ms222 (0.4 ml/l; Sigma-Aldrich, St Louis, MI, USA). Photos were taken with a Canon EOS 1200D Digital SLR Camera with EF-S 18–55 mm f/3.5–5.6 III Lens in order to follow colouration changes, characteristics of smoltification. Fish were killed by decapitation. For qPCR analyses of the tissue distribution of *tshβa* and *tshβb* transcripts, 10 fish (5 males and 5 females) were sampled in March 2015; the following organs were individually collected and stored in RNALater (Ambion Inc, Austin, USA) at −20 °C until RNA extraction: retina, brain (dissected into olfactory bulbs, telencephalon, epiphysis, optic lobes, hypothalamus, saccus vasculosus, cerebellum, medulla oblongata), pituitary, as well as samples of gill filaments, kidney, liver, spleen, muscle, skin, abdominal fat, testis or ovary. For qPCR analyses of pituitary *tshβa* and *tshβb* expression profiles throughout smoltification, 20 fish (mixed sex) were sampled once a month from February to June in 2013 and 2014, and 8 fish (mixed sex) were sampled at more frequent intervals from December 2015 to June 2016; individual pituitaries were collected in RNALater and stored at −20 °C until RNA extraction. For fluorescence *in situ* hybridization of *tshβa-* and *tshβb-*expressing pituitary cells, 8 fish were sampled in April and 8 in June 2017; individual pituitaries were collected, fixed in paraformaldehyde (PFA) overnight at 4 °C, dehydrated in increasing series of ethanol (EtOH) concentration and stored in 98% methanol at −20 °C before further processing.

### Measurement of Na^+^, K^+^-ATPase activity

Gill samples of 10 fish per sampling times were collected in experiments 2013 and 2014. For each fish, four to six primary gill filaments were placed into 100 µl of ice-cold SEI buffer (250 mM sucrose, 10 mM EDTA, 50 mM imidazole, pH 7.3) and frozen at −80 °C for measurement of gill Na^+^/K^+^-ATPase (NKA) activity. NKA activity was determined with a kinetic assay run in 96-well microplates at 25 °C and read at a wavelength of 340 nm for 10 min as described previously^54^. Gill tissue was homogenized in 150 µl of SEID (SEI buffer and 0.1% deoxycholic acid) and centrifuged at 5000 × g for 30s. Two sets of duplicate 10 µl samples were run, one set containing assay mixture and the other assay mixture and 0.5 mM ouabain. The resulting ouabain-sensitive ATPase activity is expressed as µmoles ADP mg protein^−1^ h^−1^. Protein concentrations are determined using BCA (bicinchoninic acid) Protein Assay (Pierce, www.piercenet.com, Rockford, Il, USA). Both assays were run on a THERMOmax microplate reader using SOFTmax software (Molecular Devices, www.moleculardevices.com, Menlo Park, CA, USA).

### Identification of *tshβ* paralogs in the Atlantic salmon

Gene and transcript names are in lower case and italics (e.g. *tshβ*) and protein names are in upper case (e.g. TSHβ). Atlantic salmon *tshβ* loci were identified in the recent Atlantic salmon genome assembly (ICSASG_v2, GCA_000233375.4)^24^ after interrogation of the Atlantic salmon annotated gene database in GenBank. The presence of additional *tshβ* genes was investigated by blasting salmon *tshβa* and *tshβb* against the Atlantic salmon genome. Gene sequences were examined with CLC Main Workbench 8 (Qiagen Bioinformatics, Hilden, Germany) for prediction of exons, introns, coding sequence (CDS) and signal peptide.

### Cloning and sequencing of partial cDNA of Atlantic salmon *tshβa and tshβb* paralogs

Cloning primers for Atlantic salmon *tshβa* and *tshβb* were designed on predicted mRNA sequences of corresponding genes (LOC100136355 and LOC106572976) using Primer3 (http://bioinfo.ut.ee/primer3-0.4.0/, Whitehead Institute/Massachusetts Institute of Technology, Boston, MA, US)^55^ (Supplementary Table S3). PCR was performed using cDNA of smolt pituitaries collected in April using Taq DNA polymerase (Invitrogen, Carlsbad, CA, USA) for *tshβa* and Platinum Taq DNA polymerase (Invitrogen) for *tshβb* according to the manufacturer’s instructions. Purified PCR fragments were subcloned into PCRII vectors (Thermo-Fisher, Wahtham, MA, USA) before sequencing by GATC Biotech (Brussels, Belgium). Plasmids were used for preparing cRNA probes (section fluorescence *in situ* hybridization).

### Cloning and sequencing of genomic DNA sequence of the second Atlantic salmon *tshβa*

Cloning primers for the Atlantic salmon *tshβaβ* gene were designed, based on the alignment of salmonids *tshβaβ*, on both sides of the coding sequence of the functional salmonid *tshβaβ* using Primer3 (Supplementary Table S3, Supplementary Figure x). Hatchery-produced Atlantic salmon from wild caught broodfish from the Imsa River (Norway) were used. Genomic DNA from testis samples was extracted by alkaline lysis. Testis pieces were heated in alkaline buffer (25 mM NaOH, 0.2 mM EDTA) at 95 °C for 5 min with vortexing. Reaction was stopped by adding the neutralisation buffer (40 mm Tris-HCL). PCR was performed using Atlantic salmon genomic DNA, DNA polymerase (Invitrogen), according to the manufacturer’s instructions with a thermal cycle protocol including a 10 cycle touchdown phase (from 60 to 50 °C) followed by 40 cycles with annealing temperature at 50 °C. Purified PCR fragments were subcloned into PCRII vectors (Thermo-Fisher) before sequencing by GATC Biotech.

### Illumina libraries and sequencing

Genomic DNA (gDNA) was extracted from snap-frozen liver from juvenile Loire-Allier Atlantic salmon using the Genomic-tip 100/G (Qiagen). DNA was subsequently sheared using a nebulizer (Life Technologies). Paired-end libraries were prepared from 5 μg of sheared DNA using the Paired-End Sequencing Sample Prep kit (Illumina Inc., San Diego, USA). For the library size selection step, the 400 bp band was cut from the agarose gel, purified and amplified by 10 PCR cycles. The resulting library was analyzed with a Bioanalyzer 2100 DNA 1000 series II chip (Agilent, Santa Clara, USA). All libraries were sequenced using an Illumina HiSeq2500 instrument with a read length of 2 × 151 nucleotides to a total of ~103.75 Gb of sequencing data and up to ~686.5 million reads.

### Nanopore libraries and sequencing

Genomic DNA (gDNA) was extracted from snap-frozen liver, spleen and kidney from a juvenile Loire-Allier Atlantic salmon using the Genomic tip-100/G (Qiagen). gDNA size was inspected in the TapeStation Genomic DNA system (Agilent) and found to be >60 Kb. The gDNA was subsequently sheared to 10–20 Kb fragments using a g-tube (Covaris, Woburn, MA) before library preparation. The library preparation was performed using 1D Genomic DNA by ligation for either SQK-LSK108 or SQK-LSK109 kit (Oxford Nanopore technologies, Oxford, UK). All nanopore libraries were sequenced in a FLO-MIN106 R9.4.1 SpotON Flow Cell attached to either MinION or GridION devices (Oxford Nanopore Technologies), generating 28.3 Gb of nanopore sequencing data divided over ~3.21 million reads.

### Alignment of Illumina and nanopore reads to *tshβ* paralogs

Illumina reads were aligned to the *tshb* paralogs using bowtie2 (version 2.2.5)^56^, while nanopore reads were aligned using minimap2 (version 2.5-r572)^57^ with the default Oxford nanopore parameters (map-ont). SAMtools (version 1.2)^58^ was used to remove unmapped reads from the SAM file, convert to BAM, sort and generate the index. The resulting BAM files were visualized using Integrative Genomics Viewer (IGV) (version 2.3.83)^59^.

### Phylogeny analysis

Phylogeny analysis of 39 vertebrate TSHβ amino-acid sequences was performed using a part of dataset from^21^, enriched with additional teleost TSHβ sequences, including TSHβ paralogs of salmonids identified in this study. New *tshβ* genes were either retrieved from GenBank or were identified by blasting (TBLASTN algorithm) genome assembly databases when genes were not annotated in GenBank. The amino-acid sequences of TSHβ were deduced and signal peptides were predicted using CLC Main Workbench 8 (Qiagen). The sequence alignment was performed on CLC Main workbench 8 and manually adjusted. Phylogenetic tree was constructed using Maximum Likelihood algorithm with PhyML:3.0^60^ combined to the SMS model selection^61^ and SPR as tree improvement on ATGC browser (http://www.atgc-montpellier.fr/phyml/). Tree topology was assessed by bootstrapping on 1000 replicates.

### Synteny analysis

Synteny analysis was performed on *tshβ* genomic region in actinopterygians, using a holostean, the spotted gar as a reference (LepOcu1 (GCA_000242695.1)). Comparisons were made with *tshβ* paralogons in the pike (Eluc_V3 (GCA_000721915.3)), and in two salmonid representatives, Atlantic salmon (ICSASG_v2 (GCA_000233375.4)) and rainbow trout (Omyk_1.0 (GCA_002163495.1)). Neighbouring genes of *tshβ* loci were identified and compared manually using chromosome annotation. Blast analyses on the genomes were performed to search for un-annotated genes and additional paralogs. Genes fractionated, showing a frameshift mutation or missing exon were considered pseudogenes.

### RNA extraction and cDNA synthesis

Total RNA was extracted by homogenizing tissues in TRIzol (Thermo-Fisher) according to the manufacturer’s protocol, using TissueLyser II (Qiagen). After a chloroform separation step, RNA was precipitated in ice cold isopropanol with 1 µl of glycoblue (Ambion). Total RNA was treated with DNase I (Roche Diagnostics, Basel, Switzerland) according to the manufacturer’s instructions. RNA concentration was measured using Nanopore 2000c/2000 (Thermo-Fisher). Reverse transcription was performed using 75 ng random hexamer primers (Invitrogen) and SuperScriptIII First Strand cDNA Synthesis Kit (Invitrogen) following the manufacturer’s protocol. For pituitaries, 250 ng of total RNA were used and 750 ng for brain and peripheral tissues.

### Quantitative RT-PCR

Specific quantitative real-time PCR (qPCR) primers for *tshβa* and *tshβb* were designed using Primer3 (http://bioinfo.ut.ee/primer3-0.4.0/)^55^ with forward and reverse primers on two different exons to prevent amplification of genomic DNA (Supplementary Table S3); primers were purchased from Eurofins scientific (Luxembourg). Specificity of the primers was controlled by sequencing PCR product. *β-actin* was used as reference gene using previously published primers^62^. Quantitative PCR assays were performed using LightCycler 1.2 (Roche Diagnostics) and LightCycler FastStart Master plus SYBR Green I kit (Roche Diagnostics). Each reaction contained: 4 µl of diluted cDNA template, 2 µl of SYBR green master mix and 1 µl of specific primers (500 nM final concentration). The following thermal cycling steps were used for each qPCR run: initial denaturation 94 °C for 10 min followed by 41 cycles of 10 s of denaturation at 95 °C, 5 s of annealing temperature (60 °C or 62 °C; Supplementary Table S3) and 6 s of elongation at 72 °C. The program ended by slowly increasing temperatures (0.1 °C/s) from 68–95 °C for amplification specificity controlled by melting curve analysis. Relative quantification was performed using standard curves created for each gene with serial dilutions of pooled pituitary cDNA. One dilution of the cDNA pool was added in each run as a calibrator. All samples were analysed in duplicates and each qPCR run contained a negative control using water in substitute for template cDNA. Calculations of sample concentrations were made using the Roche LightCycler 1.2 manufacturer’s software.

### Fluorescence *in situ* hybridization (FISH)

Antisense and sense cRNA probes for FISH were synthesized by *in vitro* transcription from *tshβa* or *tshβb* plasmids using T7 and SP6 RNA polymerase (Promega, Madison, Wisconsin USA) and labelled with digoxigenin-11 UTP (Roche Diagnostics) at 37 °C for 2 h. Probes were purified using Nucleospin RNA clean-UP kit (Machere-Nagal, Hoerdt, France) and controlled by gel electrophoresis. Probe length for *tshβa* was 401 bp which nearly covers the 420 bp CDS sequence. Probe length for *tshβb* was 512 bp which covers the entire CDS sequence (Supplementary Fig. S5).

Whole fixed pituitaries were rehydrated (96, 70, 50, 25% EtOH), included in 3% RNase free agarose gel and sliced into 70 µm parasagittal sections using VT1000S Leica vibratome (Leica, Wetzlar, Germany). Sections were permeabilized using proteinase K (Sigma-Aldrich, 1 µg/ml in PBS with 0.1% Tween 20, PBST) for 45 min at 37 °C, then proteinase K was inactivated using glycine (Sigma-Aldrich, 2 mg/ml in PBST) for 30 min at room temperature (RT), followed by a post fixation step in 4% PFA for 15 min and washing in PBST.

Prior to FISH, sections were incubated with hybridization buffer (HB: 50% formamide, SSC 5X, 0.1% Tween 20, 15 ng/ml Heparin, Sigma-Aldrich; 80 µg/ml Torula yeast tRNA, Sigma-Aldrich; pH 6.5) at 55 °C for 4 h. FISH was performed in fresh HB containing 300 ng/ml *tshβa* probe or 600 ng/ml *tshβb* probe at 55 °C. After 18 h, sections were washed with a series of 4 different hybridization washes (HW): HW1 (50% formamide, SSC 5× , 0.1% Tween 20) 2× 30 min, HW2 (50% HW1, 50% SSC 2× ) 2× 30 min, HW3 (SSC 2× , 1% Tween 20) 2 × 30 min, HW4 (SSC 0.2× , 0.1% Tween 20) 2 × 2 min. Sections were soaked in TNE buffer (10 mM Tris HCL pH 7.6, 500 mM NaCl, 1 mM EDTA) at 37 °C for 30 min then treated with RNaseA (Sigma-Aldrich, 20 µg/ml in TNE buffer) for 30 min at 37 °C. Sections were washed in TNE buffer, 2 × 10 min at 37 °C followed by SSC 0.2 × 0.1% Tween 20, 2 × 30 min at 55 °C. Sections were washed in PBST for 10 min with agitation at RT and incubated in PBST with 2% H2O2 for 30 min in order to inactivate endogenous peroxidases, followed by washes in PBST, 3 × 10 min with agitation. Blocking was performed using 1% Blocking Reagent (Roche Diagnostics) in Maleic acid buffer (MAB) for 2 h with agitation. Sections were incubated in blocking/MAB buffer with anti-digoxigenin peroxidase-conjugated antibody (1/250, Roche Diagnostics) overnight at 4 °C, and then washed in PBST for 2 h with agitation. Tyramide revelation was carried out using green FITC conjugated tyramide (Sigma-Aldrich, 1/200 in PBST with 0.01% H2O2) for 30 min in darkness at RT. After washing in PBST for 5 × 20 min, sections were let overnight in PBST at 4 °C in darkness. Cell nuclei were stained using DAPI staining (Sigma-Aldrich, 1/1000 in PBST) for 20 min at RT. Sections were mounted in Vectashield H-1000 Mounting Medium (Vector, Eurobio/Abcys, CA, USA). Confocal images were obtained using a confocal microscope (Zeiss LSM710, Oberkochen, Germany). Channels were acquired sequentially to avoid signal crossover between the different filters. Images were processed using the ZEN software (version 2009, Zeiss). Z**-**plan and Z**-**projection images were obtained using Image J software (Fiji software; http://rsbweb.nih.gov/ij/).

## Supplementary information

Supplementary Figures and Tables

## Data Availability

Data generated or analysed during this study are included in this article (and its Supplementary Information file).
