# Supplementary material for: Functional divergence of thyrotropin beta-subunit paralogs gives new insights into salmon smoltification metamorphosis
Source: Sci Rep. 2019 Mar 14;9:4561. doi: 10.1038/s41598-019-40019-5 (PMC6418267; doi:10.1038/s41598-019-40019-5)
Supplement: Supplementary file 1 — Supplementary Figures and Tables [file 41598_2019_40019_MOESM1_ESM.pdf]

Supplementary Information for

Functional divergence of thyrotropin beta-subunit paralogs gives new insights into salmon smoltification metamorphosis

Mitchell S. Fleming[1,2,6], Gersende Maugars[1,3,6], Anne-Gaëlle Lafont[1], Jocelyn Rancon[2], Romain Fontaine[3], Rasoul Nourizadeh-Lillabadi[3], Finn-Arne Weltzien[3], Elena Santidrian Yebra-Pimentel[4], Ron Dirks[4], Stephen D. McCormick[5], Karine Rousseau[1], Patrick Martin[2], and Sylvie Dufour[1,*]

[1]Biology of Aquatic Organisms and Ecosystems (BOREA), Muséum National d'Histoire Naturelle, CNRS, IRD, Sorbonne Université, Université de Caen Normandie, Université des Antilles, 75231 Paris Cedex 05, France

[2]Conservatoire National du Saumon Sauvage, 43300 Chanteuges, France

[3]Faculty of Veterinary Medicine, Norwegian University of Life Sciences, 0102 Oslo, Norway

[4]Future Genomics Technologies B.V. 2333 BE Leiden, Netherlands

[5]US Geological Survey, Leetown Science Center, Conte Anadromous Fish Research Laboratory, Turners Falls, MA, USA

[6]co-first authors

*Correspondence: sylvie.dufour@mnhn.fr

Supplementary Figures

```
tshβaα     ATGGAATTGTCCGTGGCCATGTGTGGTCTCCTTTGCCTGCTCTTCAGCCAAGCTGTGCCC
            M  E  S  S  V  A  M  C  G  L  L  C  L  L  F  S  Q  A  V  P
tshβaβ     ------------------------------------------------------------

tshβaα     ATGTGTGTGCCCACGGACTACACTCTGTATGAGGAGAGACGTGAATGTGACTTCTGCGTG
            M  C  V  P  T  D  Y  T  L  Y  E  E  R  R  E  C  D  F  C  V
tshβaβ     ------------------------------------------------------------

                                  Exon1        ¦         Exon2
tshβaα     GCCATCAATACGACCATTTGCATGGGCTTCTGCTACTCAAGG¦GACAGTAACATGAAGGAG
            A  I  N  T  T  I  C  M  G  F  C  Y  S  R ¦D  S  N  M  K  E
tshβaβ     ------------------------------------------¦GACAGTAATATGAAGGAG
                                                     ¦D  S  N  M  K  E

tshβaα     CTGGCCGGACCACGTTTCCTTATACAGAGAGGCTGTACCTATGACCAGGTGGAGTACCGA
            L  A  G  L  A  G  P  R  F  L  I  Q  R  G  C  T  Y  D  Q  V
tshβaβ     CTGGCTGGACCACGATTCCTGGTCCAGAGAGGCTGTACCTATGACCAGGTGGAGTACCAT
            P  V  A  P  R  F  L  V  Q  R  G  C  T  Y  D  Q  V  E  Y  H

tshβaα     ACAGTCATACTACCTGGTTGCCCACTCCATGCCAACCCTCTCTTCACCTACCCCGTAGCC
            T  V  I  L  P  G  C  P  L  H  A  N  P  L  F  T  Y  P  V  A
tshβaβ     ACAGTGATACTGCCTGGTT-CCCGCTCCATGCCAACCCTCTCTTCACCTACCCCGTAGCC
            T  V  I  L  P  G   S  R  S  M  P  T  L  S  S  P  T  P  *

tshβaα     CTCAGCTGCCACTGTGGCACCTGCAACACAGACAGTGATGAGTGTGCCCACAAGGCCAGC
            L  S  C  H  C  G  T  C  N  T  D  S  D  E  C  A  H  K  A  S
tshβaβ     CTCAGCTGCCACTGTGGCAGTTGCAACACAGACAGTGATGAGTGTGCCCACAAGGCCAGC

tshβaα     AGTGGAGACGGCGCCAGGTGTTCCAAGCCACTCAGACACATCTACC-ATACCCTGGCCTG
            S  G  D  G  A  R  C  S  K  P  L  R  H  I  Y   H  T  L  A  *
tshβaβ     AGTGGTGACAGCCCCAGGTCTACCAAACTACTCAGACACATCTACCCATAGCCTGGCCTG

tshβaα     A-----------------------
tshβaβ     AACAACTACATCCACCCCAACTAA
```

Figure S1A. *Tshβa* paralogs in salmonid species. Figure S1A. Sequence alignment of *Salmo salar tshβaα* and *tshβaβ* paralogs. Coding sequences and amino-acid sequences of *Salmo salar tshβaα* [19] and *tshβaβ* (LOC106566072; and this study) are aligned. Exon 1 is missing in *tshβaβ*. A nucleotide deletion (-) in *tshβaβ* exon 2 leads to an early stop codon (TAG). *Salmo salar tshβaβ* sequence was confirmed by cloning genomic sequence in a Norwegian specimen and by Illumina and Nanopore sequencing of the genome of a Loire-Allier specimen, supporting that *tshβaβ* is a pseudogene in both populations.

Ref 19: see reference list in the article.

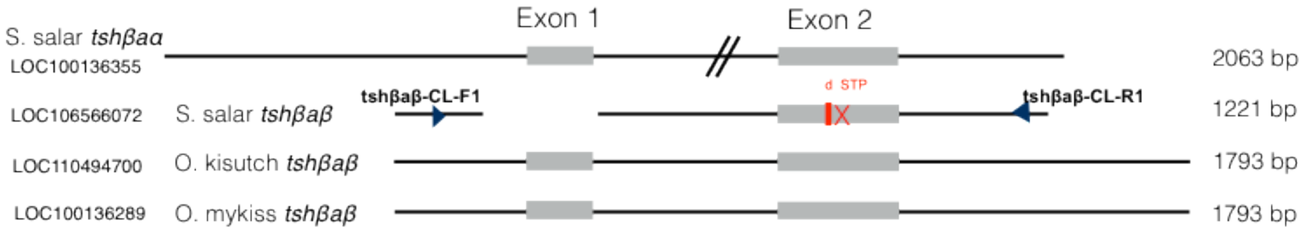

Figure S1B. Gene structure of *tshβaα* and *tshβaβ* paralogs in *Salmo salar* and *Oncorhynchus sp.* *Salmo salar tshβaα* gene is composed of two exons as well as *Oncorhynchus sp. tshβaα* (not shown) and *tshβaβ* genes. *Salmo salar tshβaβ* gene is missing exon 1 and presents a deletion (d) in exon 2 which leads to an early stop codon (STP). Sequences of primers used for cloning the genomic sequence of *tshβaβ* **(**arrow**)** are provided in Table S2.

```
A. anguilla TSHβb    M--ALDSLACVLLCLLLGQALAKCVPQNYTLYVEREGCEHCVAVNTTVCRGFCFSRDTNMKKCGL-KGFPVQRACMYQSLVYHAVSLPGC
S. formosus TSHβb    M--TVSPFASGILCLMMIWAVFACAPKNYTLYVQRLHCDRCVAINTTVCSGFCYSQDTNLRGQMG-RWQPYQRGCTYQMLAYQTAVLPGC
D. rerio TSHβb       M----RVLLCSFLLLLGEDALLACSLKNYTLYVEKHECGHCMAINTTVCSGMCFTRDTNVQGFVG-KRFLLQQSCMHRSLVYRSARMPGC
P. nattereri TSHβb  M--GFVALPHILLFFSAAGVLLGCSLRNFTLHVEKPECGRCMVINTTICSGMCFSQDSNLRGLMG-RAFLIQAVCVYQSVEYRSVKLPGC
E. lucius TSHβb*    M----CVLMWLLLCVLMGGGMCGCMTENYTLHIEKSGCNQCVTINTTICSGFCHTQDTNVKGRVG-RSYLIQRGCMPHTLVHRAARVPGC
S. salar TSHβb      M----YVLAWVLLFVWLGGGVCVCMMENYTLLIEKRGCSQCIAVNTTICSGFCHTQDTNVKGRVG-KSYLIQRGCMPHSLVYHPARVPGC
O. kisutch TSHβb    M----YALTWVLLFVWLGGGVSVCMMENYTLLIEKRACSQCIAVNTTICSGFCHTQDTNVKGRVG-KSYLIQRGCMPHSLVYHPARVPGC
O. mykiss TSHβb     M----YALTWVLLFVWLGGGVSVCMMENYTLLIEKRGCSQCIAVNTTICSGFCHTQDTNVKGRVG-KSYLIQRGCMPHSLVYHPARVPGC
G. morhua TSHβb     M--SL--FVCVLLCVLIADTACGCTLKNFTLMIEKYECEQCVLINTTICSGYCYTQDTNFRGRVG-KNFLIQRGCTPGSLVYRTARLVGC
O. niloticus TSHβb  M--PLSALKSLLLCALMVGAVYTCMLKNYTLWIEKQDCTQCVAINTTICSGYCYTQDTNLKGRFG-RTFLIQRSCVPLSLVYQAAHIPGC
O. latipes TSHβb    M--SLFMLKSALVLAVMAGTVCACVLKNHTIWVEKQNCTQCIAINTTICSGYCYSRDTNFRGRFG-RTFLIQRSCMPLSLVYRVAHIPGC
D. labrax TSHβb     M--PSLALKCMLLCTLTGWTVCACMLKNHTLWIERHDCAQCVAVNTTICSGYCYTQDTNLRGRFG-RTFLIQRSCVPLSLVYRPARLPGC
G. aculeatus TSHβb  M--PLLVLKCMLLCALMHRAACACMLSNHTLWIESRDCAQCVAINTTICKGYCYTKDTNLKGRFG-RDFMIQRSCVPLSLVYRAVHLPGC
A. anguilla TSHβa   M--RVVLLASAVLCLLAGQVLSICSPVDYTLYVEKPECDFCVAINTTICMGFCYSLDPNVVGPAVKR-LVVQRGCTYQAVEYRTAELPGC
S. formosus TSHβa   MGSSTLALTCGLLYMLGGRALSLCSLTDYTLYVEKPGCDYCVAINTTICMGFCYSWDTNMVGLVGKR-LLLQRGCTYRSIEYQTITLPGC
D. rerio TSHβa      M--SL-LYVIGMLGLLMKVAVPMCAPTDYTIYIERQECNYCVAVNTTICMGFCFSRDSNIKELVGPR-FIVQRGCTYQEVEYRTAVLPGC
P. nattereri TSHβa  M--SAAVLVAGILGLLLGSAMPMCTPTEYTLYIEKQECDYCVAINTTICMGFCFSRDSNMKELVGPR-FLIQRSCTYQEVEYRTAALPGC
E. lucius TSHβa     M--ESSVVMCGL-CLLFSQAVTICVPNEYTLYVEKQECDFCVAINTTICMGFCYSRDSNMREMAGPR-FLIQRGCTYNKVEYRTVTLPGC
S. salar TSHβa      M--ELSVAMCGLLCLLFSQAVPMCVPTDYTLYEERRECDFCVAINTTICMGFCYSRDSNMKELAGPR-FLIQRGCTYDQVEYRTVILPGC
O. kisutch TSHβaα   M--ELSVAMCGLLCLLFSQAVPMCVPTDYTLYEERRECDFCVAINTTICMGFCYSRDSNMKELAGPR-FLIQRGCTYDQVEYRTVILPGC
O. mykiss TSHβaα    M--ELSVAMYGLLCLLFSQAVPMCVPTDYTLYEERRECDFCVAINTTICMGFCYSRDSNMKELAGPR-FLIQRGCTYDQVEYRTVILPGC
O. kisutch TSHβaβ   M--ESSVAMCGLLCLLFSQAVPMCVPTDYTLYEERHECDFCVAINTTICMGFCYSRDSNMKELAGPR-FLVQRGCTYDQVEYRTVILPGC
O. mykiss TSHβaβ    M--ESSVAMCGLLCLLFSQAVPMCVPTDYTLYEERHECDFCVAINTTICMGFCYSRDSNMKELAGPR-FLVQRGCTYDQVEYRTVILPGC
G. morhua TSHβa     M--DYFVFVGSVLLLMFSPAAPMCVPTDYTLYVEKPECNFCVAINTTICMGFCYSRDSNIGDLVGLR-FLLQRGCTYNQVEYRTAILPGC
O. niloticus TSHβa  M--EATVFNCWLFFLMFSPAVPMCLPTDFTLYVEKPECEFCVAINTTICMGFCYSRDSNMRDILGPR-FLVQRGCTYDKVEYHTAILPGC
O. latipes TSHβa    M--NTVLFPFWMLFLLLSPVVPMCLPTDFTLYVEKPECDYCVAVNTTICTGFCYSRDSNMRDIFGPR-FLIQRGCTYDKVEYRSAILPGC
D. labrax TSHβa     M--ETAVFSCWLLFLLFSPAVPMCLPTDFTLYVERPECDFCVAINTTICMGFCYSRDSNMRDILGPR-FLIQRGCTYDKVEYRTAILPGC
G. aculeatus TSHβa  M--ETAVFPCWLLFLLLSPAVPTCFPTDFTMYVERPECDYCVAINTTICMGFCYSRDSNVRAIVGPR-FLIQTGCNYDKVEYRAALLPGC
L. oculatus TSHβa   M--GAALLVCGLLCLVASQTLSKCAPTDYMLYVEKYGCAYCVAINTTICSGFCYSRDTNVKGVVG-KSYFLQRSCTYQVLEYRTVLLPGC
H. sapiens TSHβ     M--TALFLMSMLFGLTCGQAMSFCIPTEYTMHIERRECAYCLTINTTICAGYCMTRDINGK-LFLPKYALSQDVCTYRDFIYRTVEIPGC
S. vulgaris TSHβ    M--SPFFVMSLLLGLIFGQTASLCAPSEYTIHVEKRECAYCLAINTTICAGFCMTRDSNGKKLLL-KSALSQNVCTYKEMLYRTALIPGC
C. mydas TSHβ       M--SPIFLMSLLFGLTFGQAMSFCAPIEYIIHVEKRECAYCLAINTTICAGFCMTRDSNGKKLLL-KSALSQNVCTYKDMVYRTVVLPGC
N. parkeri TSHβ     M--TSVFMVSFLLCFAYGHAALLCMLTEYTMYVEKEECAYCIAVNTTICSGYCQTKDPNMKR-SLPEINLNQNICTYSDYIHKTVSVPGC
L. chalumnae TSHβ   M--NHICLVSILLYLVVRQALSICSLTQHTIYVEKRECTSCLVVNTTICSGYCRTRDVIMKNRLLPKTALSQHVCTYKDIEYNSVTVPGC
C. milii TSHβ       M-SSRLLLLILFLCGGRAH--PYCSPSPYLQYLEQDQCEFCLVINTTICSGSCLTRDANVK-RLLPKSALSQNICTFDELEYRTVRIPGC
L. chalumnae TSHβ2  M-NFTWLVPVVICMSC-TSVNSLCTVTRYMMYVEKEQCSHCIAINTTICSGYCITRDPNLKA-LLPRTALSQSVCTYNKVKYLTIRIPGC
C. milii TSHβ2      M-NAMWLLPLVLCLSG-SQIGFTCSLTRHVVYVEKEECSYCMAINTTVCAGYCMSRDVNIKT-LLPKNALVQNVCTFHNIRYMMIRLPGC
R. typus TSHβ2      M-NTLQLLALILSLSC-HRVVSQCSLTRFVSYVEKEECSHCLAINTTGCAGFCISRDVNTKS-LLPKIALIQRVCTYQDVKYISIKLPGC
P. marinus GpHβ     M-GPLQLFQLALWLEV-AYSNSLCKLHNTTIAVEKSGCAECRYINTTVCSGYCYTW----QLIGHNMRKIAQEVCTYTDVGYETVTLHGC

A. anguilla TSHβb    PPDVDPLFSFPVALRCHCSRCNTSNTECLHRGKRLPNPCDSTLCYAKGPPKAATATSLTGTYQENQKMGEAAVIYQEV*-------
S. formosus TSHβb    QLNVDSLYSYPAALSCHCARCDTASSDCIHKVKDITR-ANTSSCLANHTYDLQLHSH------QPQTHLHTHLGSN*---------
D. rerio TSHβb       PVHIDPLFFYPVARRCNCTKCNTSRNECVFRHK-----HKHNRC-SK----------------QLRTV*-----------------
P. nattereri TSHβb  PAHADPLFVYPIARGCHCSKCNTVRNECVH-TL-----RRSHTCRLK----------------QLQTTDQ*---------------
E. lucius TSHβb*    PLHISPLLYFPEVHRCHCTRCDGHAHRCVHKAQD-----TPAPCPRTSPSP-----------------------------------
S. salar TSHβb      PLHANNVLYYPESRRCHCTRCDGHTHRCVHVTQV-----TPTPCTRKNPATHRTSSSTRRTWKRPPVKKNSDQET*----------
O. kisutch TSHβb    PLHVNNVLYYPESRRCHCTRCDGHAHRCVHMTQA-----TPTPCTRKSPAT-------RRTWTRPPVKKHSDQET*----------
O. mykiss TSHβb     PLHVNNVLYYPESRRCHCTRCDGHAHRCVHMTQA-----TPTPCTRKSPAT-------RRTWTRPPVKKHSDQET*----------
G. morhua TSHβb     PRNVNPVIYYPEFHRCKCRSCDRRTHHCVQKSRY-----PLNQC-RKTR-----------------HKRKGKDSN*---------
O. niloticus TSHβb  PKDVNPQLYYPAAHCCSCRRCDTRTHRCVRTSRI-----PYDQCFTTLDSVKK----------QNQSALEISQYSNTSGSALARK*
O. latipes TSHβb    PPDVNAELYYPVAHCCSCRCCDTRTYHCVQPRRF-----SYDQCSVKLGSGGH----------QNENCFGNITNC*----------
D. labrax TSHβb     PHGVDPQLYYPAARRCSCRRCDTRTHHCVRTSRI----SSYYRCSTGLKGSVGS---------QKQPHALGN*-------------
G. aculeatus TSHβb  PPGVNPQVYYPAAHRCLCKRCDTRTHHCVRTSRV-----STEGCSATLDGVK-SQT-------QPSVATQQYVNTGDAA*------
A. anguilla TSHβa   PPHVDPRFSYPVALHCTCRACDPARDECTHRASA-----DGDRCSKPLLLH---------MHAYPGQSNYIQTL*-----------
S. formosus TSHβa   QRHANPLFSYPVAQDCYCSTCDTGSHECTHKGAG----DSSVQCAKPLLHI----------YPYPGQSNHV*--------------
D. rerio TSHβa      PSHADPHFTYPVALSCHCSTCKTHSDECALRTRS-----AGMRCSKPVHHL----------YPE--ENNYAQAYWDQ-YE*-----
P. nattereri TSHβa  PPHADPNFTYPVALSCHCSMCNTRSDECSHKSSR-----SAVKCSRPVRHL----------YPYPIESDLIQPGWLQMLE*-----
E. lucius TSHβa     PLRADPLFTYPVALSCHCGTCNTNSDECAHKAGS----GDGARCSKPLRHV----------FPYAGLNNYIHPN*-----------
S. salar TSHβa      PLHANPLFTYPVALSCHCGTCNTDSDECAHKASS----GDGARCSKPLRHI----------YHTLA*-------------------
O. kisutch TSHβaα   PLHANPLFTYPVALSCHCGTCNTDSDECAHKASS----GDGARCSKPLRHI----------YP*----------------------
O. mykiss TSHβaα    PLHANPLFTYPVALSCHCGTCNTDSDECAHKASS----GDGARCSKPLRHI----------YPYPGLNSYIHPN*-----------
O. kisutch TSHβaβ   PLHANPLFTYPVALSCHCGTCNTDSDECAHKASS----GDGPRCTKPLRNI----------YPYPGLSNYIHPN*-----------
O. mykiss TSHβaβ    PLHANPLFTYPVALSCHCGTCNTDSDECAHKASS----GDGPRCTKPLRHI----------YPYPGLNNYIHPN*-----------
G. morhua TSHβa     PSEGSSLFSYPVALSCHCGACNTAVDECAHRASS----NR-PTCTKPVRHI----------Y----QSNFLLPF*-----------
O. niloticus TSHβa  PIEANPVFTYPVALSCHCSACRTDTDECAHRA-S----MDGTKCTKPVRRI----------YPYPGHSNYVIPF*-----------
O. latipes TSHβa    PLESNPVFTYPVALSCHCSACRTDSDECTHRA-S----TGGGRCTKPVRLV----------HPYPGQSTYMILF*-----------
D. labrax TSHβa     PLDANPMFTYPVALSCHCGACRTDSDECAHRA-S----VDGTRCTKPVRRL----------YPYPGQTNYMIPF*-----------
G. aculeatus TSHβa  PIDSDPVFSYPVALSCRCGTCRTDSDECVHRAPG----VGGARCTKPVRRI----------YPYPGQSTYMTPF*-----------
L. oculatus TSHβ    PLHINPLLSYPLALRCHCSRCNTDNNDCTHKA------SETNECTKPIQPA----------DSYPGQSNYIQLD*-----------
H. sapiens TSHβ     PLHVAPYFSYPVALSCKCGKCNTDYSDCIH------EAIKTNYCTKPQK-------------------SYLVGFSV*---------
S. vulgaris TSHβ    PHHTVPYYSYPVAVSCKCGKCNTDYSDCVR------ERLRTNYCTKPQK-------------------LCNL*-------------
C. mydas TSHβ       PRHTVSYYSYPVATNCKCGKCNTDYSDCIH------ETVRTDYCTKPQK-------------------PYNV*-------------
N. parkeri TSHβ     PMHVNSLYTYPVALSCRCDKCNTDYIDCVQ------DRIESNYCTKPRM-------------PKDFLYNYAKNIIRQRFK*-----
L. chalumnae TSHβ   PPHVSPHYSYPVARSCSCDKCNTDYTDCTH------ETVRTNHCIKPTV------------------ASPLKLF*-----------
C. milii TSHβ       PTGVSSQHSYPTALSCKCKNCDTDYTDCTVQ-----ENLEANVCRKPQ----------------SETNSQD*--------------
L. chalumnae TSHβ2  APHIDPYYRFAVAINCKCSLCNTDSTDCTNEGE------NPNECNQPQWR-------------IPAMKSRLLLI*-----------
C. milii TSHβ2      PPDIDPFYRLPVVLSCQCSQCATETTDCTNDIAN----QNPYHCTKPQWR-------------IPATNSRIFIL*-----------
R. typus TSHβ2      PPNVDPFYRLPVILSCVCSQCTTDTTDCINGV-N-----ASFDCTKPQWS-------------IPISRSRILPLSGNV*-------
P. marinus GpHβ     DPGVDPTLHYPVALSCQCSQCQTDTTDCTVRS------LRPDYCSHPSQIKGPPLGVDLTNETVPAAGSYRV*-------------
```

Figure S2. Alignment of TSHβ sequences. The alignment displays amino-acid sequences of gnathostome TSHβ and of lamprey glycoprotein hormone β-subunit, used for phylogeny analysis (Fig. 1). The amino-acid sequences were deduced from genomic sequences, alignment was performed and signal peptides predicted, using CLC Main Workbench 8. Sequence references are provided in Supplementary Table S1. Signal peptide sequences are highlighted in green, cysteine residues are in red, and conserved potential N-glycosylation sites in blue. *: indicates partial sequence.

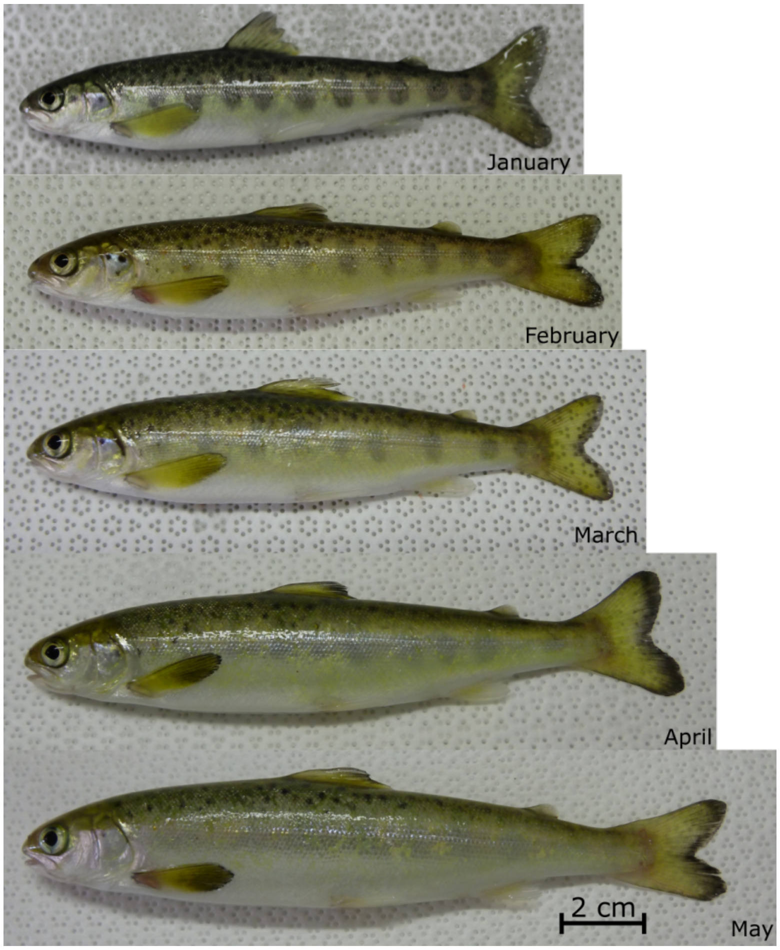

Figure S3. Colouration changes during smoltification. All Loire-Allier Atlantic salmon sampled in 2016 experiment were photographed and the figure displays the characteristic morphological changes of smoltification. Dark parr marks on the dorsal lateral side were highly visible in parr (January) and regressed throughout experimental period. Conversely, progressive silvering of the belly region occurred as well as darkening of the pectoral fins throughout experimental period. All these changes are typical of smoltification.

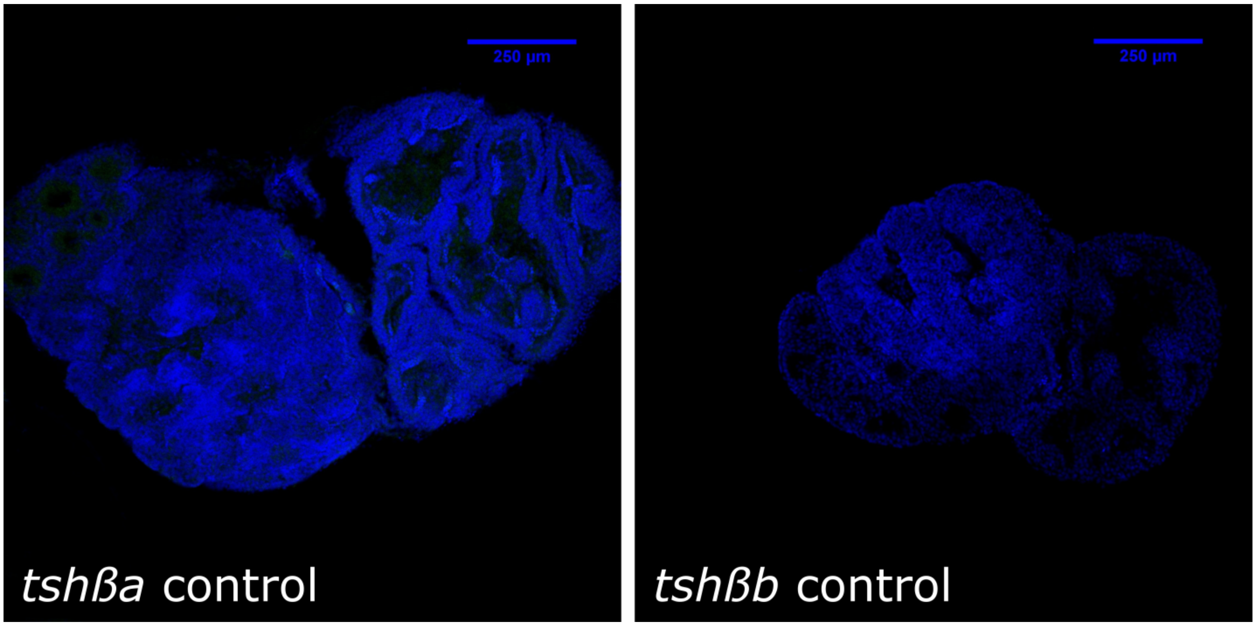

Figure S4. Control fluorescent *in situ* hybridization. Fluorescent *in situ* hybridization (FISH) with sense probes of Atlantic salmon *tshβa* and *tshβb* was performed on 70 µm parasagittal sections of pituitaries of smolts sampled in April 2017. Photos: Confocal z-plan images. No *tshβa* and *tshβb* labelling was observed (green, FITC); cell nuclei are labelled in blue (DAPI).

Atlantic salmon (*Salmo salar*) *tshβa* mRNA (AF060566,[19])
CDS – 420bp
Probe primers:
F1 - gtctcctttgc ctgctcttc
R1 - agttgttcag gccagggta
Probe length 401bp

```
gattgtgtga cggaacatct tcgctgtttg ccaaatggaa ttgtccgtgg ccatgtgtgg
tctcctttgc ctgctcttca gccaagctgt gcccatgtgt gtgcccacgg actacactct
gtatgaggag agacgtgaat gtgacttctg cgtggccatc aatacgacca tttgcatggg
cttctgctac tcaagggaca gtaacatgaa ggagctggcc ggaccacgtt tccttataca
gagaggctgt acctatgacc aggtggagta ccgaacagtc atactacctg gttgcccact
ccatgccaac cctctcttca cctaccccgt agccctcagc tgccactgtg gcacctgcaa
cacagacagt gatgagtgtg cccacaaggc cagcagtgga gacggcgcca ggtgttccaa
gccactcaga cacatctacc ataccctggc ctgaacaact acatcccccc caactgataa
cgctctccct tccctagata gtttggctgt aatgaccata atgaccaata aggcaaggag
aatggtcagt tcaaatcaag tttatggtca gtagcctaat cattatgata acagaacagc
attgttgtat tcatgtgttt gtggtggata cagtgagcta cagggtggtg tatcctattc
attgtgacat gtattgttgc tgttgtgtgt ggtatgttaa tatgcctttt tgtttgctac
tttgcttgca ttggaggctg atcctggtat ttctgtctgc ctcatgtctt tcctgtccat
tgattcactg agctattttg tttacggtga accaatatca tgaatgggac gttattaaag
ctgaagtctg taaaaaaaaa aaaaaa
```

Atlantic salmon (*Salmo salar*) *tshβb* mRNA (MG948546, this study)
CDS – 468
Probe primers:
F1 - agtgtacgag ttgtgttcca g
R1 - cg ctgtgtggat aggcagttgg
Probe length 512bp

```
agtgtacgag ttgtgttcca gtatgtatgt gttggcgtgg gtgctgctgt ttgtgtggct
gggtggaggt gtgtgcgtgt gtatgatgga aaactacacg ctactgatag agaagagagg
ttgttctcag tgtattgccg tcaacaccac catctgcagt ggcttctgcc acacacagga
caccaatgtg aaggggcgtg tgggtaagag ttacctgatc caacgtggct gcatgcccca
ctccctggtc tatcatcccg cccgcgtgcc aggctgcccg ctgcatgcca acaacgtgct
ctactaccca gagtcccgcc gctgccactg cacccgctgt gacggacacg cccaccgctg
tgtccacgtg acccaggtca cacccacccc ttgcaccagg aagaaccctg ccacccacag
gaccagctcc tcaacccgca ggacctggaa acgcccacct gtcaagaaaa acagcgacca
ggaaacctaa ccaactgcct atccacacag cgaccag
```

Figure S5. *Salmo salar tshβa* and *tshβb* mRNA sequences and probes used for FISH (fluorescence *in situ* hybridization). The CDS sequences are highlighted in yellow. The forward and reverse primers used for preparing the FISH probes are indicated in red.

Supplementary Tables

Sequence references						
Scientific name	Common name	Gene	Accession number	Reference	Locus / Genomic location	Genome assembly
Anguilla anguilla	*European eel*	*tshβa*	CAA51908.1	Salmon et al. 1993 (ref 63)	X73493	
		tshβb	CDX47224	Maugars et al. 2014 (ref 21)	LM655248	
Callorhinchus milii	*elephant shark*	*tshβ*	XM_007901614.1	Dos Santos et al., 2011 (ref 64)	LOC103183879	Callorhinchus_milii-6.1.3 (GCF_000165045.1)
		tshβ2	XM_007895400.1	Dos Santos et al., 2011 (ref 64)	LOC103179889	
Chelonia mydas	*green sea turtle*	*tshβ*	XM_007066210.1		LOC102944542	CheMyd_1.0 (GCF_000344595.1)
Danio rerio	*zebrafish*	*tshβa*	XM_021476792		LOC353223	GRCz11 (GCF_000002035.6)
		tshβb	XM_017353619		LOC100001596	
Dicentrarchus labrax	*European sea bass*	*tshβa*	CBN80754.1 / HG916837	Kuhl et al., 2011 (ref 65)	(23152376..23152537,23153440..23153718)	CBXY01 (GCA_000689215.1)
		tshβb	HG916841		(11140731..11140892,11141262..11141540)	
Esox lucius	*northern pike*	*tshβa*	XM_010866556		LOC105007569	Eluc_V3 (GCF_000721915.3)
		tshβb	GATF01036761.1			
Gadus morhua	*Atlantic cod*	*tshβa*	CAEA01180393	Tørresen et al., 2017 (ref 66)	(29..295,573..734)	gadMor2
		tshβb	CAEA01110666		(1511..1768,1904..2083)	
Gasterosteus aculeatus	*three-spined stickleback*	*thsβb*	AANH01002636		(56644..56925,57107..57268))	GCA_000180675.1
		tshβa	AANH01007474		(80149..80310,80748..81047)	
Homo sapiens	*Human*	*Tshβ*	NP_000540.2	Keita et al., 1988 (ref 67)	LOC7252	GRCh38.p12 (GCF_000001405.38)
Latimeria chalumnae	*coelacanth*	*tshβ*	XM_005988466		LOC102363810	LatCha1 (GCF_000225785.1)
		tshβ2	XM_006002575		LOC102362870	
Lepisosteus oculatus	*spotted gar*	*tshβ*	XM_006628383		LOC102690058	LepOcu1 (GCF_000242695.1)
Nanorana parkeri	*Tibetan frog Nanorana parkeri*	*tshβ*	XM_018570902.1		LOC108799084	ASM93562v1 (GCF_000935625.1)
Oncorhynchus kisutch	*coho salmon*	*tshβaα*	XM_020482112		LOC109890149	Okis_V1 (GCF_002021735.1)
		tshβaβ	XM_020494167		LOC109899110	
		tshβb	XM_020474930		LOC109882835	
Oncorhynchus mykiss	*rainbow trout*	*tshβaα*	NM_001124543	Ito et al., 1993 (ref 68)	LOC100136289	Omyk_1.0 (GCF_002163495.1)
		tshβaβ	XM_021569992		LOC110494700	
		tshβb	XM_021614507		LOC110531299	
Oreochromis niloticus	*Nile tilapia*	*tshβa*	XM_003453648		LOC100534562	ASM185804v2 (GCA_001858045.2)
		tshβb	XM_005478141		LOC102082579	Orenil1.1 (GCF_000188235.2)
Oryzias latipes	*Japanese medaka*	*tshβa*	XM_004068796		LOC101171658	ASM223467v1 (GCF_002234675.1)
		tshβb	XM_011477157		LOC105354380	
Petromyzon marinus	*sea lamprey*	*gphβ2*	AY730276.1			
Pygocentrus nattereri	*red piranha*	*tshβa*	XM_017697768		LOC108427544	Pygocentrus_nattereri-1.0.2 (GCF_001682695.1)
		tshβb	XM_017693267		LOC108424941	
Rhincodon typus	*whale shark*	*tshbβ2*	XM_020524860		LOC109924889	ASM164234v2 (GCF_001642345.1)
Salmo salar	*Atlantic salmon*	*tshβaα*	NM_001123528 / AF060566	Martin et al., 1999 (ref 19)	LOC100136355	ICSASG_v2 (GCF_000233375.1)
		tshβaβ			LOC106566072	
		tshβb	XM_014147573 / MG948546		LOC106572976	
Scleropages formosus	*Asian arowana*	*tshβa*	XM_018725541		LOC108918388	ASM162426v1 (GCF_001624265.1)
		tshβb	XM_018760281		LOC108939147	
Sturnus vulgaris	*Common starling*	*tshβ*	XM_014872791		LOC106851066	Sturnus_vulgaris-1.0 (GCF_001447265.1)

Table S1. References of sequences used for alignment (Figure S1) and phylogeny (Figure 1). The table provides species names, gene names and gene sequence references.

Ref 19, 21: see reference list in the article.

Ref 63. Salmon, C., Marchelidon, J., Fontaine, Y. A., Huet, J. C., & Querat, B. Cloning and sequence of thyrotropin beta subunit of a teleost fish: the eel (Anguilla anguilla L.). *Comptes rendus de l'Académie des Sciences. Série III, Sciences de la vie*, *316*(8), 749-753 (1993).

Ref 64. Dos Santos, S., Mazan, S., Venkatesh, B., Cohen-Tannoudji, J., & Quérat, B. Emergence and evolution of the glycoprotein hormone and neurotrophin gene families in vertebrates. *BMC Evolutionary Biology*, *11*(1), 332 (2011).

Ref 65. Kuhl, H., Tine, M., Hecht, J., Knaust, F., & Reinhardt, R. Analysis of single nucleotide polymorphisms in three chromosomes of European sea bass Dicentrarchus labrax. *Comparative Biochemistry and Physiology Part D: Genomics and Proteomics*, *6*(1), 70-75 (2011).

Ref 66. Tørresen, O. K., *et al*. An improved genome assembly uncovers prolific tandem repeats in Atlantic cod. *BMC genomics*, *18*(1), 95 (2017).

Ref 67. Keita, T., Yoshihide, H., Yoshiki, H., Kiyoshi, M., & Kenichi, M. The structure of the human thyrotropin β-subunit gene. *Gene*, *73*(2), 489-497(1988).

Ref 68. Ito, M., Koide, Y., Takamatsu, N., Kawauchi, H., & Shiba, T. cDNA cloning of the beta subunit of teleost thyrotropin. *Proceedings of the National Academy of Sciences*, *90*(13), 6052-6055 (1993).

Gene	Gene description	Spotted gar (*Lepisosteus oculatus*)		Pike (*Esox lucius*)					
		LG3 (NC_023181.1)		LG17 (NC_025984.3)		NW_017858932		LG12 (NC_025979.3)	
		Gene ID	Position	Gene ID	Position	Gene ID	Position	Gene ID	Position
ap4b1	adaptor related protein complex 4 beta 1 subunit	102684786	26964117..26976083					105030751	1026827..1044557
rplp2	ribosomal protein lateral stalk subunit P2	102692126	29040501..29043346			105008159	256438..259606	105009991	196768..199029
kcna10	potassium voltage-gated channel subfamily A member 10	102689659	29095866..29097648			105008161	213177..215462		
ngf	nerve growth factor	102692738	29313557..29353194			105007566	50964..90970	105028952	401287..440872
tspan2	tetraspanin 2	102689859	29378082..29423571			105007567	18307..31253	105028940	448524..469085
slc25a22	mitochondrial glutamate carrier 1-like	102692936	29425713..29433964			105007568	12477..17289	105028941	470213..481023
tshβ	thyroid stimulating hormone beta	102690058	29437049..29478708			105007569	8381..9944		484522..486383
slc16a1	solute carrier family 16 member 1	102684984	26979839..27017991	1,05E+08	33324027..33343181			105028950	644155..663054

Gene	Atlantic Salmon (*Salmo salar*)							
	ssa22 (NC_027321.1)		ssa12 (NC_027311.1)		ssa15 (NC_027314.1)		ssa13 (NC_027312.1)	
	Gene ID	Position	Gene ID	Position	Gene ID	Position	Gene ID	Position
ap4b1					106572995	103033835..103068830		
rplp2	100194651	8474485..8483767	106566032	78938666..78948735	106572979	102776661..102779529		
kcna10	106582604	8575779..8577626	106566035	78899016..78905068	106572980	102797234..102799006	106566322	1606573..1608553
ngf	106582728	8696713..8720733	106566077	78741742..78774738			106566305	542002..542964
tspan2	106582727	8734680..8751055	106566039	78710403..78722990	106572998	103276744..103306031		
slc25a22	106582726	8751131..8764328	106566040	78700651..78707563				
tshβ	100136355	8772597..8774381	106566072	78686748..78687716	106572976	102662737..102664183		
slc16a1	106582732	8821693..8834407	106566043	78608935..78629165			106566331	2328437..2336930

Gene	Rainbow trout (*Oncorhynchus mykiss*)							
	17 (NC_035093.1)		7 (NC_035083.1)		9 (NC_035085.1)		16 (NC_035092.1)	
	Gene ID	Position	Gene ID	Position	Gene ID	Position	Gene ID	Position
ap4b1					110532865	67238584..67240300		
rplp2	110494710	65934306..65941003	110527753	31913133..31924688	110532870	67642016..67645327		
kcna10	110494706	65885166..65888930	110527755	31975841..31978536	110531301	67654466..67656250	110492706	69822208..69824549
ngf			110527108	32125486..32129846				
tspan2	110494702	65704884..65716391	110527760	32140714..32159374	110531298	67488874..67495011		
slc25a22	110494701	65695740..65702753	110527759	32158946..32174930				
tshβ	110494700	65681650..65683616	100136289	32179881..32181851	110531299	67507231..67508041		
slc16a1	110494698	65621102..65638206	110527763	32232338..32244729			110492722	70493736..70495918

Table S2. References of genes used for synteny analysis (Figure 2). The table provides gene name description, Gene ID and position (start and end) for *tshβ* and its neighbouring genes used for the synteny analysis (Figure 2). Chromosome or scaffold numbers are highlighted in grey. Each gene is single in the spotted gar (used as a reference) while they are represented by up to 2 paralogs in the pike and up to 4 paralogs in the Atlantic salmon and rainbow trout.

Gene Name	Primer name	Primer Sequence	Annealing Temperature	Primer Reference
PCR-Cloning (CL)				
tshβa AF060566	tshβa-CL-F	GTCTCCTTTGC CTGCTCTTC	55°C	This paper
	tshβa-CL-R	AGTTGTTCAG GCCAGGGTA	55°C	This paper
tshβb MG948546	tshβb-CL-F	AGTGTACGAG TTGTGTTCCA G	55°C	This paper
	tshβb-CL-R	CG CTGTGTGGAT AGGCAGTTGG	55°C	This paper
tshβaβ LOC10656607	tshβaβ-CL-F	TGGTGCAGCCTTGCTGAAGGACC	Touchdown	This paper
	tshβaβ-CL-R	AGCCATCCTACAGATGCAGGGTCT	Touchdown	This paper
qPCR				
tshβa AF060566	tshβa-F	CTCCTTTGCCTGCTCTTCAG	60°C	This paper
	tshβa-R	GGCCAGCTCCTTCATGTTAC	60°C	This paper
tshβb MG948546	tshβb-F	TTGCCGTCAACACCACCAT	62°C	This paper
	tshβb-R	GGGATGATAGACCAGGGAGTG	62°C	This paper
β-actin	actin-F	CCAAAGCCAACAGGGAGAAG	60°C	[62]
	actin-R	AGGGACAACACTGCCTGGAT	60°C	[62]

Table S3. Primer sequences. The table provides the sequences of Forward and Reverse primers used for PCR of Atlantic salmon *tshβa*, *tshβb* (cDNA cloning and FISH) and *tshβaβ* (genomic cloning) and for qPCR of Atlantic salmon *tshβa*, *tshβb* and *β-actin*.
